# Supplementary material for: 2-Butyl-2-tert-butyl-5,5-diethylpyrrolidine-1-oxyls: Synthesis and Properties
Source: Molecules. 2020 Feb 14;25(4):845. doi: 10.3390/molecules25040845 (PMC7070904; doi:10.3390/molecules25040845)
Supplement: Supplementary file 1 [file molecules-25-00845-s001.pdf]

2-Butyl-2-tert-butyl-5,5-diethylpyrrolidine-1-oxyls: Synthesis and properties

Irina F. Zhurko[1], Sergey Dobrynin[1], Artem A. Gorodetskii[1], Yuri I. Glazachev[2], Tatyana V. Rybalova[1], Elena I. Chernyak[1], Nargiz Asanbaeva[1,3], Elena G. Bagryanskaya[1,*] and Igor A. Kirilyuk[1,*]

[1] Novosibirsk Institute of Organic Chemistry, Siberian Branch of Russian Academy of Sciences, Academician Lavrentiev Ave. 9, Novosibirsk 630090, Russia; zhurko@nioch.nsc.ru (I.Z.), s.a.dobrynin@gmail.com (S.D.); gorodaa@nioch.nsc.ru (A.G.), rybalova@nioch.nsc.ru (T.R.), chernyak@nioch.nsc.ru (E.C.), nargiz-asan@mail.ru (N.A.), egbagryanskaya@nioch.nsc.ru (E.B.), kirilyuk@nioch.nsc.ru (I.K.)

[2] Institute of Chemical Kinetics & Combustion SB RAS, Institutskaya 3, Novosibirsk 630090, Russia; glaza@ngs.ru (Y.G.)

[3] Novosibirsk State University, 2 Pirogova St., Novosibirsk 630090, Russia

* Correspondence: kirilyuk@nioch.nsc.ru, egbagryanskaya@nioch.nsc.ru; Tel.: +7 (383) 330-8850 (E.B.)

Supporting Information

Table of Contents

1. IR spectral data. S5

1.1 5,5-Diethyl-1-hydroxy-2,2,4-trimethyl-2,5-dihydro-1*H*-imidazol (**7**) (**Fig. SI1**). S5

1.2 (Z)-1-(5,5-Diethyl-1-hydroxy-2,2-dimethylimidazolidin-4-ylidene)-3,3-dimethylbutan-2-one (**9**) (**Fig. SI2**). S5

1.3 (Z)-4-(3,3-Dimethyl-2-oxobutylidene)-5,5-diethyl-2,2-dimethylimidazolidin-1-oxyl (**10**) (**Fig. SI3**). S6

1.4 5-(*tert*-Butyl)-2,2-diethyl-3-oxo-3,4-dihydro-2*H*-pyrrole 1-oxide (**6**) (**Fig. SI4**). S6

1.5 3-Amino-5-(*tert*-butyl)-2,2-diethyl-2*H*-pyrrole 1-oxide (**13**) (**Fig. SI5**). S7

1.6 5-(*tert*-Butyl)-2,2-diethyl-3-imino-4-oxo-3,4-dihydro-2*H*-pyrrole 1-oxide (**16**) (**Fig. SI6**). S7

1.7 5-(*tert*-Butyl)-2,2-diethyl-3,4-dioxo-3,4-dihydro-2*H*-pyrrole 1-oxide (**17**) (**Fig. SI7**). S8

1.8 3,3'-Bis(2-*tert*-butyl-5,5-diethyl-4-oxopyrrolinylidene) 1,1'-dioxide (**12**) (**Fig. SI8**). S8

1.9 5-(*tert*-Butyl)-5-butyl-2,2-diethyl-3-oxopyrrolidin-1-oxyl (**18**) (**Fig. SI9**). S9

1.10 1-(*tert*-Butoxy)-2,2,6,6-tetramethylpiperidine (**20**) (**Fig. SI10**). S9

1.11 3,3'-bis(2-Butyl-5,5-diethyl-4-oxopyrrolinylidene) 1,1'-dioxide (**21**) (**Fig. SI11**). S10

1.12 5-(*tert*-Butyl)-5-butyl-2,2-diethyl-3-hydroxypyrrolidin-1-oxyl (**4**) (**Fig. SI12**). S10

1.13 5-(*tert*-Butyl)-2,2-diethyl-3,4-bis(methoxycarbonyl)pyrrolidine (**23a,b**) (**Fig. SI13, SI14**). S11

1.14 5-(*tert*-Butyl)-2,2-diethyl-3,4-bis(hydroxymethyl)-3,4-dihydro-2*H*-pyrrole 1-oxide (**24**)(**Fig. SI15**). S12

1.15 2-(*tert*-Butyl)-2-butyl-5,5-diethyl-3,4-bis(hydroxymethyl)pyrrolidin-1-oxyl (**5**) (**Fig. SI16**). S12

1.16 5-(*tert*-Butyl)-2,2-diethyl-3-(hydroxymethyl)-4-pentyl-3,4-dihydro-2*H*-pyrrole (**26**) (**Fig. SI 17**). S13

1.17 3-Carboxy-2,2,5,5-tetraethylpyrrolidin-1-oxyl (**1**) (**Fig. SI18**) and its' sodium salt (**1Na**) (**Fig. SI19**). S13

1.18 2,2,5,5-Tetraethyl-3,4-bis(hydroxymethyl)pyrrolidin-1-oxyl (**2**) (**Fig. SI20**). S14

2. UV spectral data. S15

2.1 5,5-Diethyl-1-hydroxy-2,2,4-trimethyl-2,5-dihydro-1*H*-imidazol (**7**) (**Fig. SI21**). S15

2.2 (Z)-1-(5,5-Diethyl-1-hydroxy-2,2-dimethylimidazolidin-4-ylidene)-3,3-dimethylbutan-2-one (**9**) (**Fig. SI22**). S15

2.3 (Z)-4-(3,3-Dimethyl-2-oxobutylidene)-5,5-diethyl-2,2-dimethylimidazolidin-1-oxyl (**10**) (**Fig. SI23**). S16

2.4 5-(*tert*-Butyl)-2,2-diethyl-3-oxo-3,4-dihydro-2*H*-pyrrole 1-oxide (**6**) (**Fig. SI24**). S16

2.5 5-(*tert*-Butyl)-2,2-diethyl-3,4-dioxo-3,4-dihydro-2*H*-pyrrole 1-oxide (**17**) (**Fig. SI25**). S17

2.6 3,3'-bis(2-*tert*-Butyl-5,5-diethyl-4-oxopyrrolinylidene) 1,1'-dioxide (**12**) (**Fig. SI26**). S17

2.7 5-(*tert*-Butyl)-5-butyl-2,2-diethyl-3-oxopyrrolidin-1-oxyl (**18**) (**Fig. SI27**). S18

2.8 3,3'-bis(2-Butyl-5,5-diethyl-4-oxopyrrolinylidene) 1,1'-dioxide (**21**) (**Fig. SI28**). S18

2.9 5-(*tert*-Butyl)-5-butyl-2,2-diethyl-3-hydroxypyrrolidin-1-oxyl (**4**) (**Fig. SI29**). S19

2.10 5-(*tert*-Butyl)-2,2-diethyl-3,4-bis(hydroxymethyl)-3,4-dihydro-2*H*-pyrrole 1-oxide (**24**) (**Fig. SI30**). S19

2.11 2-(*tert*-Butyl)-2-butyl-5,5-diethyl-3,4-bis(hydroxymethyl)pyrrolidin-1-oxyl (**5**) (**Fig. SI31**). S20

2.12 5-(*tert*-Butyl)-2,2-diethyl-3-(hydroxymethyl)-4-pentyl-3,4-dihydro-2*H*-pyrrole (**26**) (**Fig. SI32**). S20

3. NMR (^{1}H and $^{13}C\{1H\}$) spectral data of diamagnetic compounds. S21

3.1 5,5-Diethyl-1-hydroxy-2,2,4-trimethyl-2,5-dihydro-1*H*-imidazol (**7**) (**Fig. SI33, SI34**). S21

3.2 (Z)-1-(5,5-Diethyl-1-hydroxy-2,2-dimethylimidazolidin-4-ylidene)-3,3-dimethylbutan-2-one (**9**) (**Fig. SI35, SI36**). S22

3.3 1.4 5-(*tert*-Butyl)-2,2-diethyl-3-oxo-3,4-dihydro-2*H*-pyrrole 1-oxide (**6**) (**Fig. SI37, SI38**). S23

3.4 3-Amino-5-(*tert*-butyl)-2,2-diethyl-2*H*-pyrrole 1-oxide (**13**) (**Fig. SI39, SI40**). S24

3.5 5-(*tert*-Butyl)-2,2-diethyl-3-imino-4-oxo-3,4-dihydro-2*H*-pyrrole 1-oxide (**16**) (**Fig. SI41, SI42**). S25

3.6 5-(*tert*-Butyl)-2,2-diethyl-3,4-dioxo-3,4-dihydro-2*H*-pyrrole 1-oxide (**17**) (**Fig. SI43, SI44**). S26

3.7 3,3'-Bis(2-*tert*-butyl-5,5-diethyl-4-oxopyrrolinylidene) 1,1'-dioxide (**12**) (**Fig. SI45, SI46, SI47**). S27

3.8 1-(*tert*-Butoxy)-2,2,6,6-tetramethylpiperidine (**20**) (**Fig. SI48, SI49**). S28

3.9 3,3'-Bis(2-butyl-5,5-diethyl-4-oxopyrrolinylidene) 1,1'-dioxide (**21**) (**Fig. SI50, SI51**). S29

3.10 5-(*tert*-Butyl)-2,2-diethyl-3,4-bis(methoxycarbonyl)pyrrolidine (**23a,b**) (**Fig. SI52-SI55**). S30

3.11 5-(*tert*-Butyl)-2,2-diethyl-3,4-bis(hydroxymethyl)-3,4-dihydro-2*H*-pyrrole 1-oxide (**24**) (**Fig. SI56, SI57**). S32

3.12 5-(*tert*-Butyl)-5-butyl-2,2-diethylpyrrolidine-1,3-diol 2,2,2-trifluoroacetate (**4H**) (**Fig. SI58**). S33

3.13 (2-(*tert*-Butyl)-2-butyl-5,5-diethyl-1-hydroxypyrrolidine-3,4-diyl)dimethanol 2,2,2-trifluoroacetate (**5H**) (**Fig. SI59**). S34

3.14 3-Carboxy-2,2,5,5-tetraethyl-1-hydroxypyrrolidin-1-ium 2,2,2-trifluoroacetate (**1H**) (**Fig. SI60**). S34

3.15 (3S,4S)-2,2,5,5-Tetraethyl-1-hydroxy-3,4-bis(hydroxymethyl)pyrrolidin-1-ium 2,2,2-trifluoroacetate (**2H**) (**Fig. SI61**). S35

4. 1D and 2D NMR spectra used for structure determination of compound **26** (5-(*tert*-butyl)-2,2-diethyl-3-(hydroxymethyl)-4-pentyl-3,4-dihydro-2*H*-pyrrole). S36

4.1 ^{1}H NMR spectrum of **26** (**Fig. SI62**). S36

4.2 ^{13}C NMR spectrum of **26** (**Fig. SI63**). S36

4.3 ^{1}H-^{1}H (COSY) NMR spectrum of **26** (**Fig. SI64**). S37

4.4 ^{1}H-^{13}C (HSQC) NMR spectrum of **26** (**Fig. SI65**). S37

4.5 ^{1}H-^{13}C (HMBC) NMR spectrum of **26** (**Fig. SI66**). S38

4.6 Assignment of the signals in NMR spectra of compound **26**. (**Table SI1**) S38

5. HPLC UV data for **1**, **2**, **4** and **5**. S40

5.1 5-(*tert*-Butyl)-5-butyl-2,2-diethyl-3-hydroxypyrrolidin-1-oxyl (**4**) (**Fig. SI67**). S40

5.2 2-(*tert*-Butyl)-2-butyl-5,5-diethyl-3,4-bis(hydroxymethyl)pyrrolidin-1-oxyl (**5**) (**Fig. SI68, SI69**). S40

5.3 3-Carboxy-2,2,5,5-tetraethylpyrrolidin-1-oxyl (**1**) (**Fig. SI70**). S41

5.4 2,2,5,5-Tetraethyl-3,4-bis(hydroxymethyl)pyrrolidin-1-oxyl (**2**) (**Fig. SI71**). S42

6. X-ray crystallography data. S43

6.1 Experimental details for compounds **4**, **5**, **12** (**Table S2**). S43

6.2 The structure and atom numbering of **4** (**Fig. SI72**). S44

6.3 The structure and atom numbering of **5** (**Fig. SI73**). S45

6.4 The structure and atom numbering of **12**) (**Fig. SI74**). S45

7. EPR spectral data (**Fig. SI75**). S46

8. Reduction kinetics data (**Fig. SI76, SI77**). S47

9. Thermal stability of nitroxides **4** and **5** (**Fig. SI78**). S48

1. IR spectral data

1.1 5,5-Diethyl-1-hydroxy-2,2,4-trimethyl-2,5-dihydro-1*H*-imidazol (**7**).

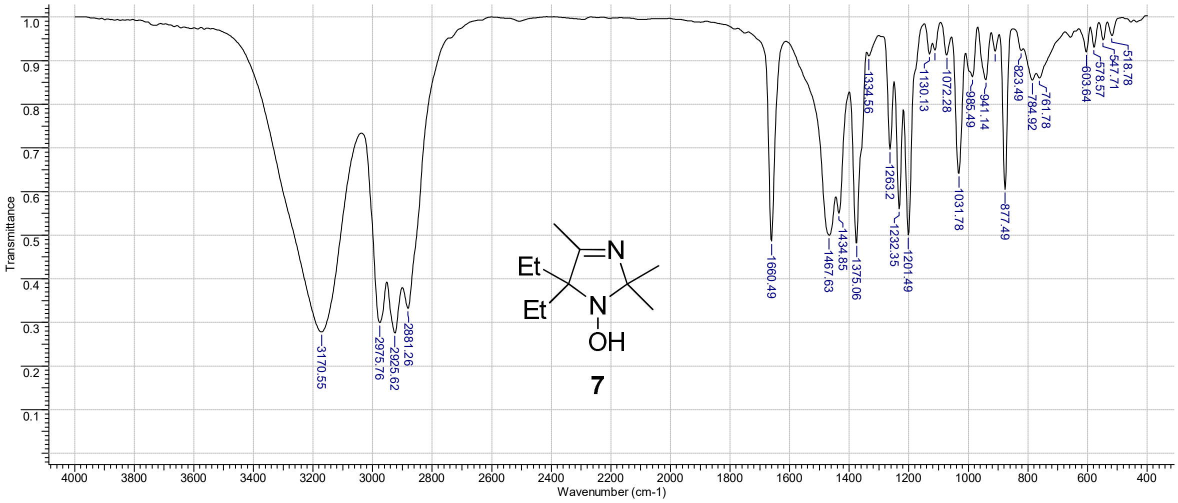

Figure SI1. IR spectrum of **7** (KBr).

1.2 (Z)-1-(5,5-diethyl-1-hydroxy-2,2-dimethylimidazolidin-4-ylidene)-3,3-dimethylbutan-2-one (**9**)

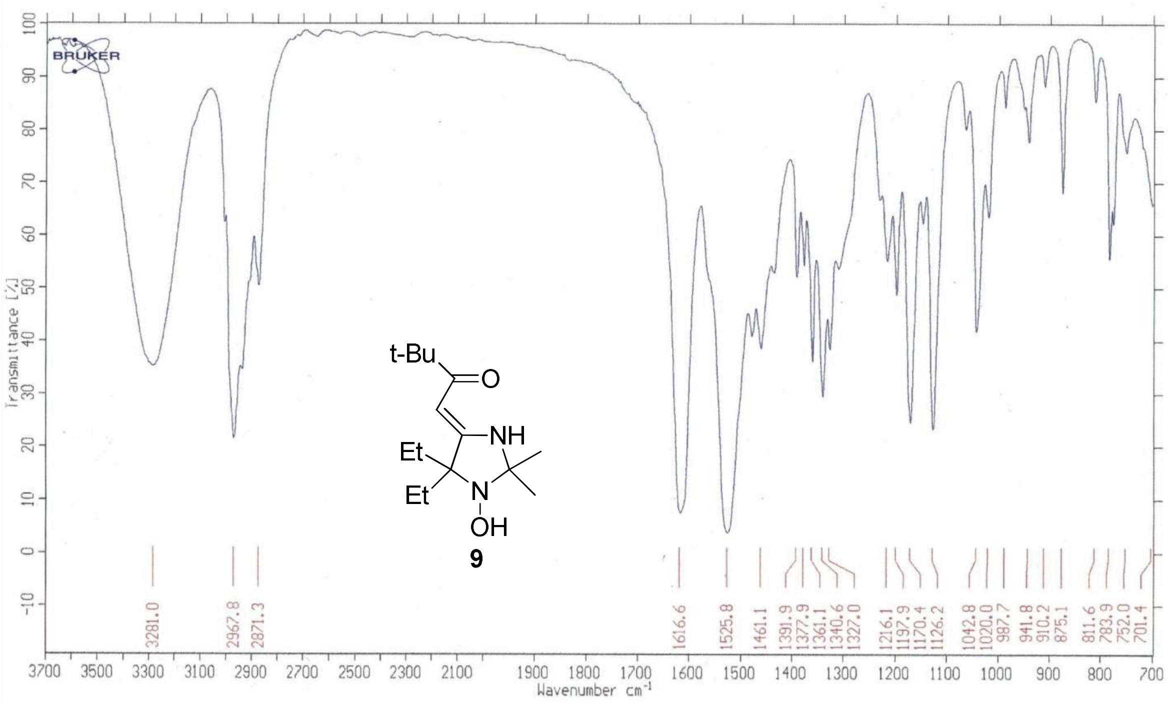

Figure SI2. IR spectrum of **9** (KBr).

1.3 (Z)-4-(3,3-dimethyl-2-oxobutylidene)-5,5-diethyl-2,2-dimethylimidazolidin-1-oxyl (**10**)

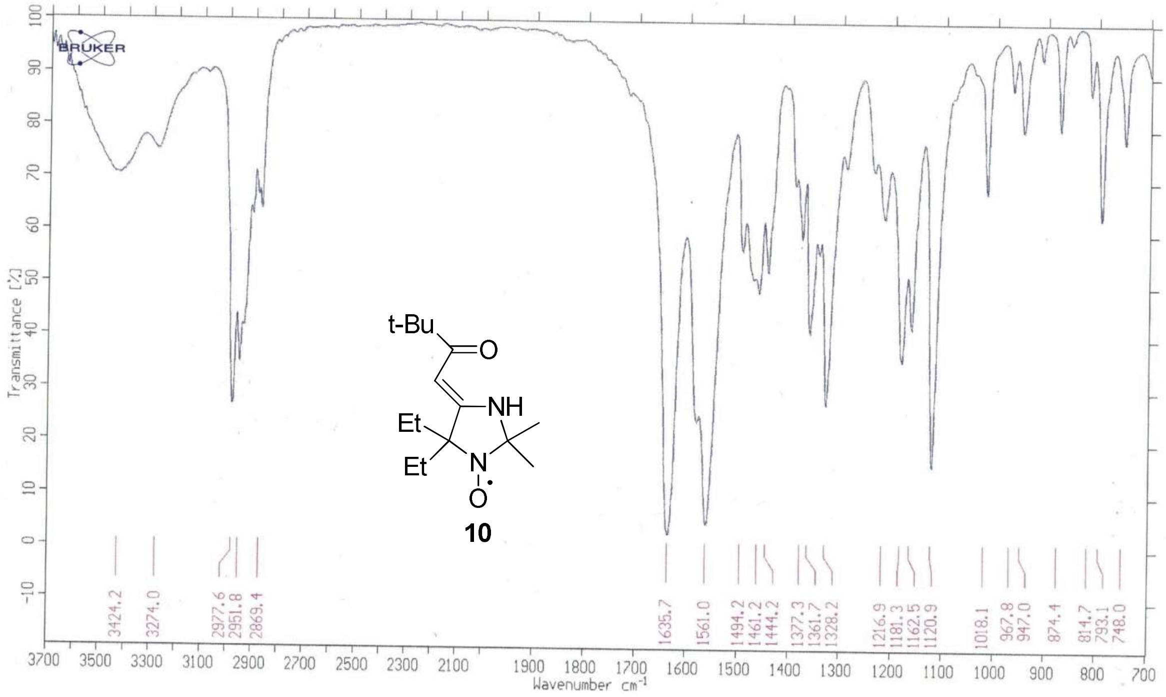

Figure SI3. IR spectrum of **10** (KBr).

1.4 5-(*tert*-Butyl)-2,2-diethyl-3-oxo-3,4-dihydro-2*H*-pyrrole 1-oxide (**6**)

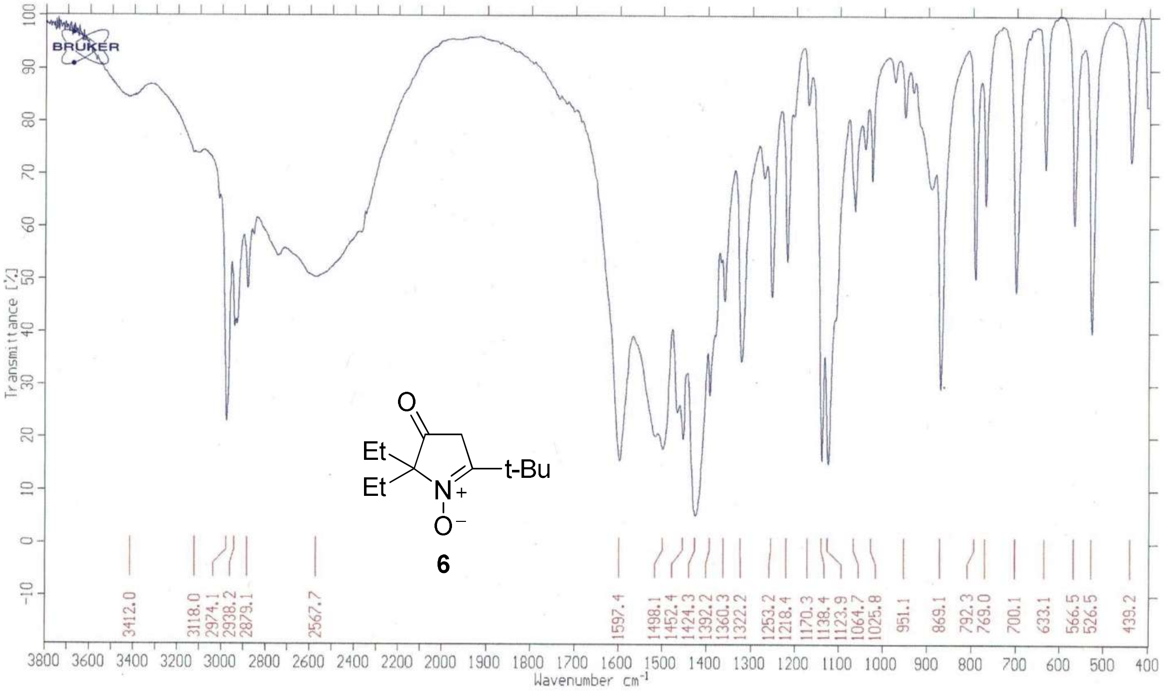

Figure SI4. IR spectrum of **6** (KBr).

1.5 3-Amino-5-(*tert*-butyl)-2,2-diethyl-2*H*-pyrrole 1-oxide (**13**)

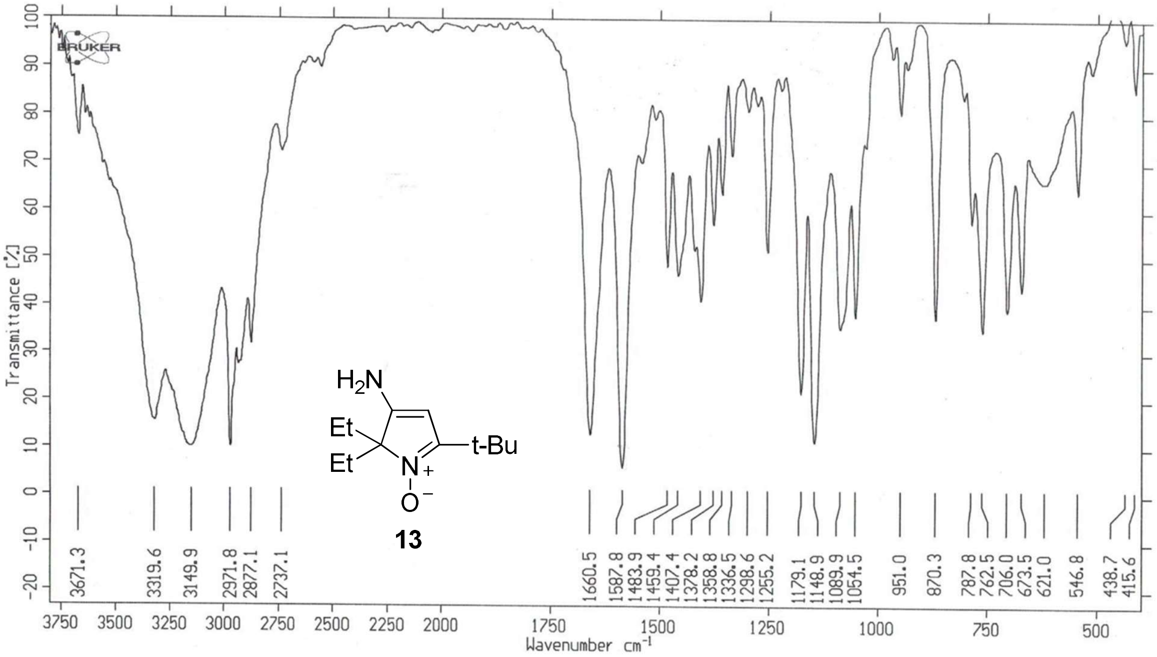

Figure SI5. IR spectrum of **13** (KBr).

1.6 5-(*tert*-Butyl)-2,2-diethyl-3-imino-4-oxo-3,4-dihydro-2*H*-pyrrole 1-oxide (**16**)

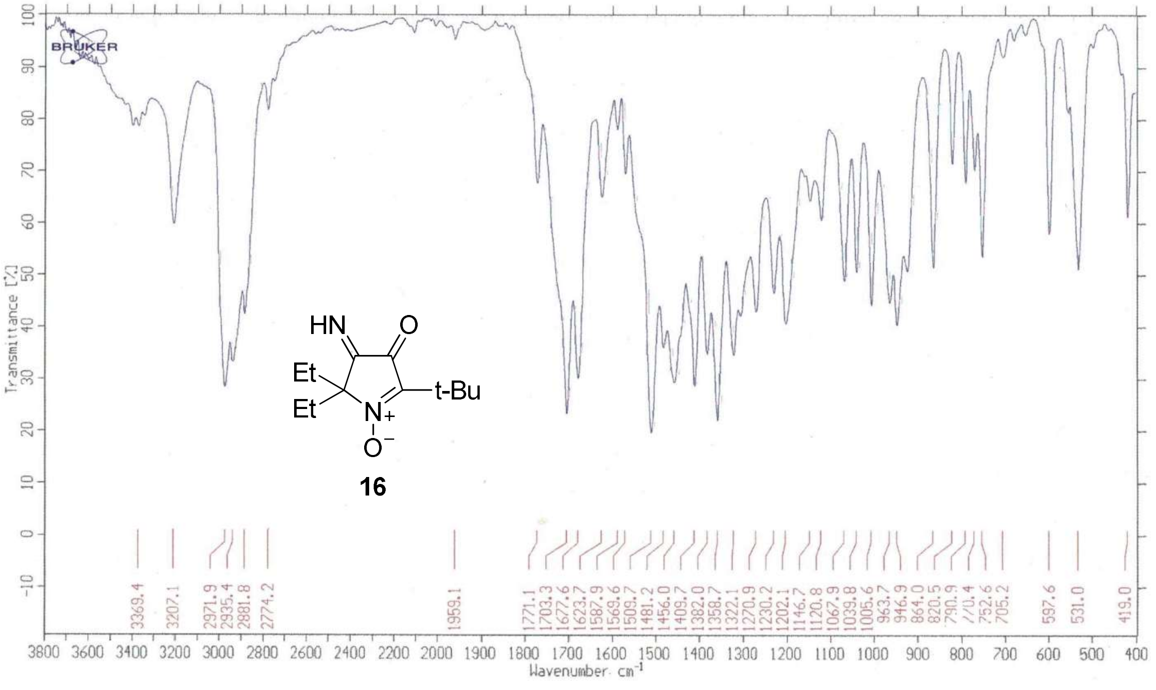

Figure SI6. IR spectrum of **16** (neat).

1.7 5-(*tert*-Butyl)-2,2-diethyl-3,4-dioxo-3,4-dihydro-2*H*-pyrrole 1-oxide (**17**)

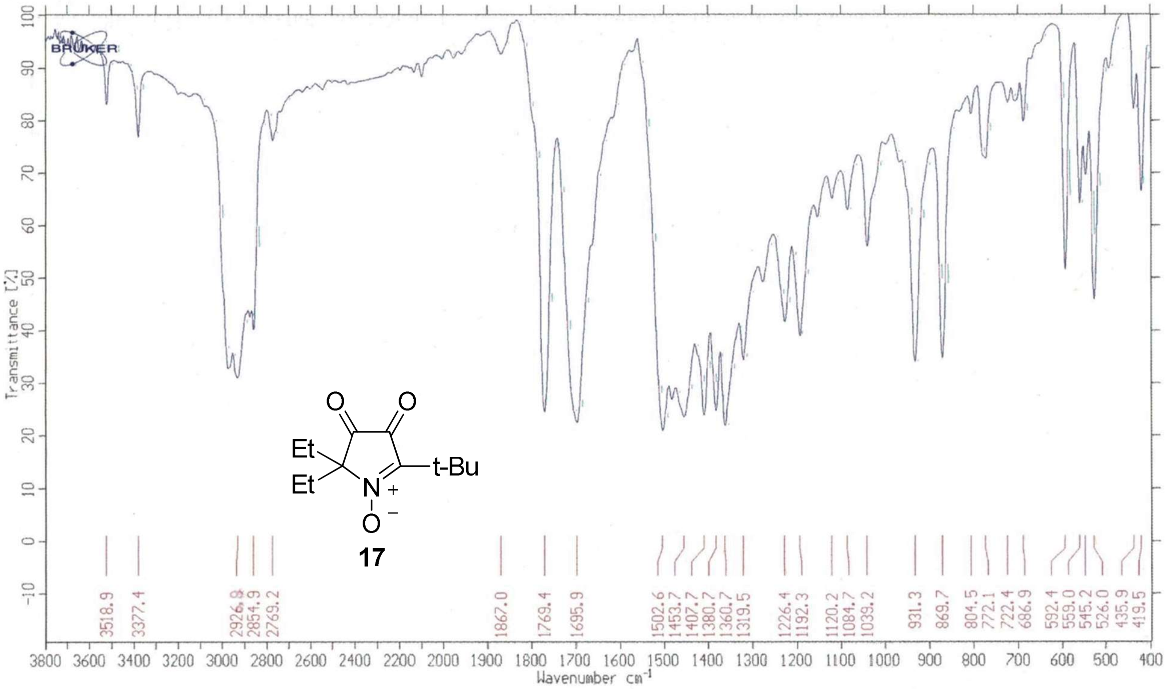

Figure SI7. IR spectrum of **17** (KBr).

1.8 3,3'-bis(2-*tert*-Butyl-5,5-diethyl-4-oxopyrrolinylidene) 1,1'-dioxide (**12**)

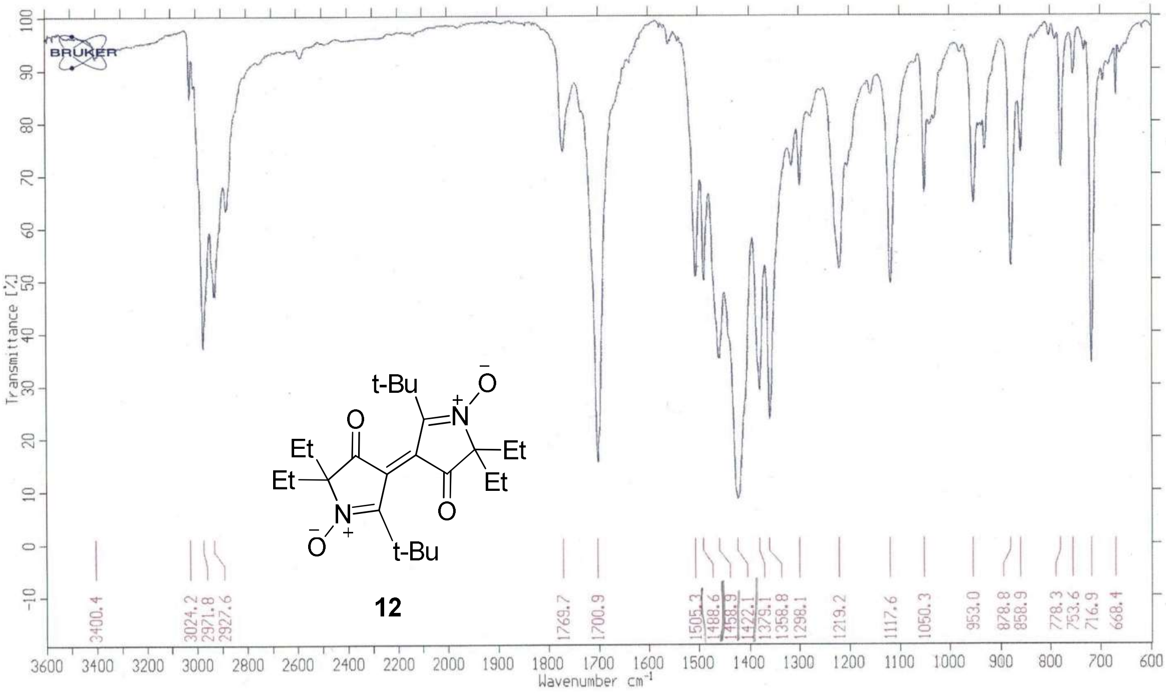

Figure SI8. IR spectrum of **12** (KBr).

1.9 5-(*tert*-Butyl)-5-butyl-2,2-diethyl-3-oxopyrrolidin-1-oxyl (**18**)

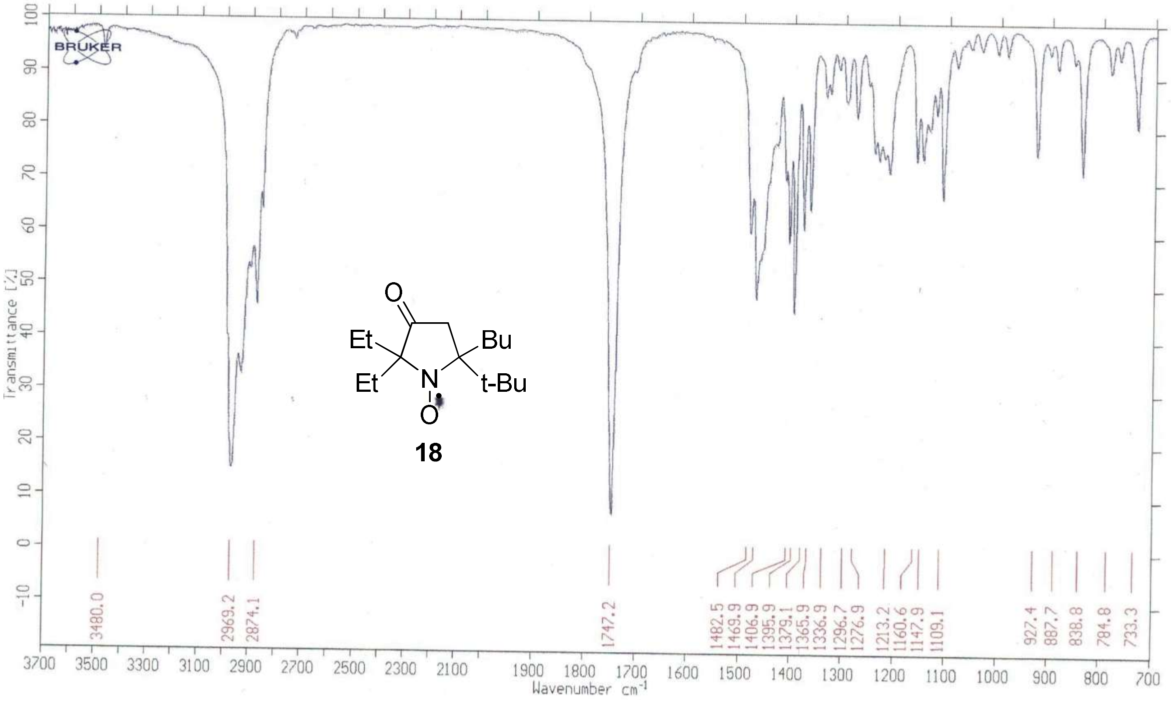

Figure SI9. IR spectrum of **18** (KBr).

1.10 1-(*tert*-Butoxy)-2,2,6,6-tetramethylpiperidine (**20**)

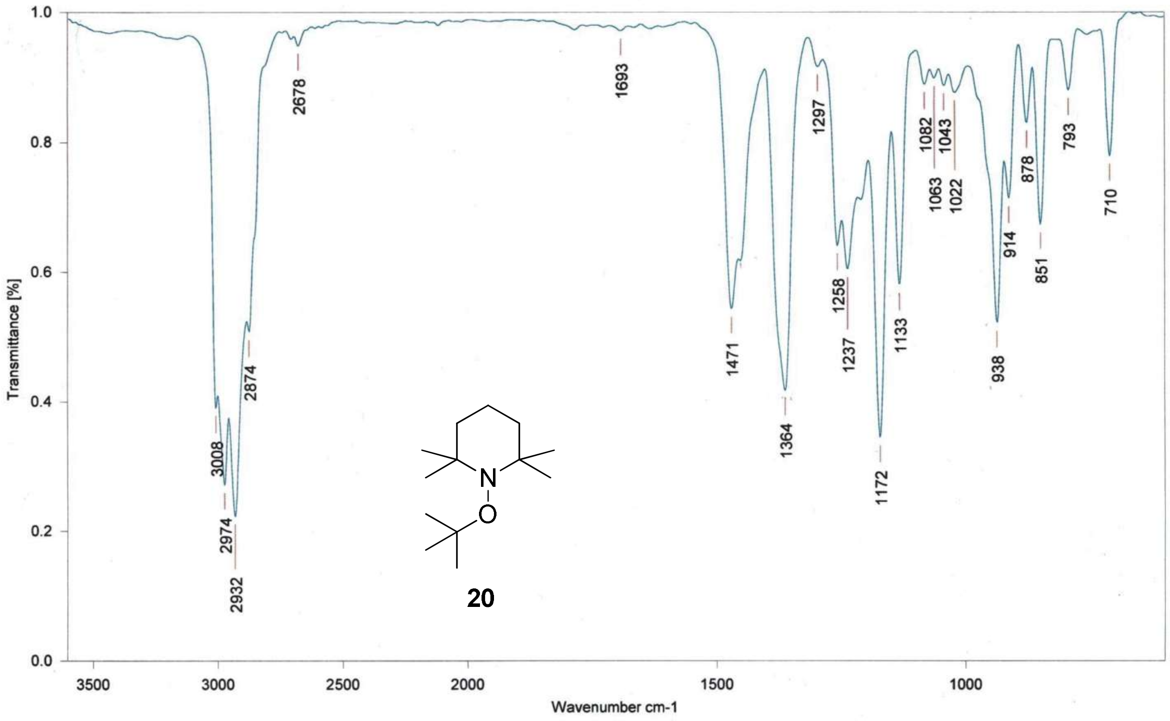

Figure SI10. IR spectrum of **20** (neat).

1.11 3,3'-bis(2-Butyl-5,5-diethyl-4-oxopyrrolinylidene) 1,1'-dioxide (**21**)

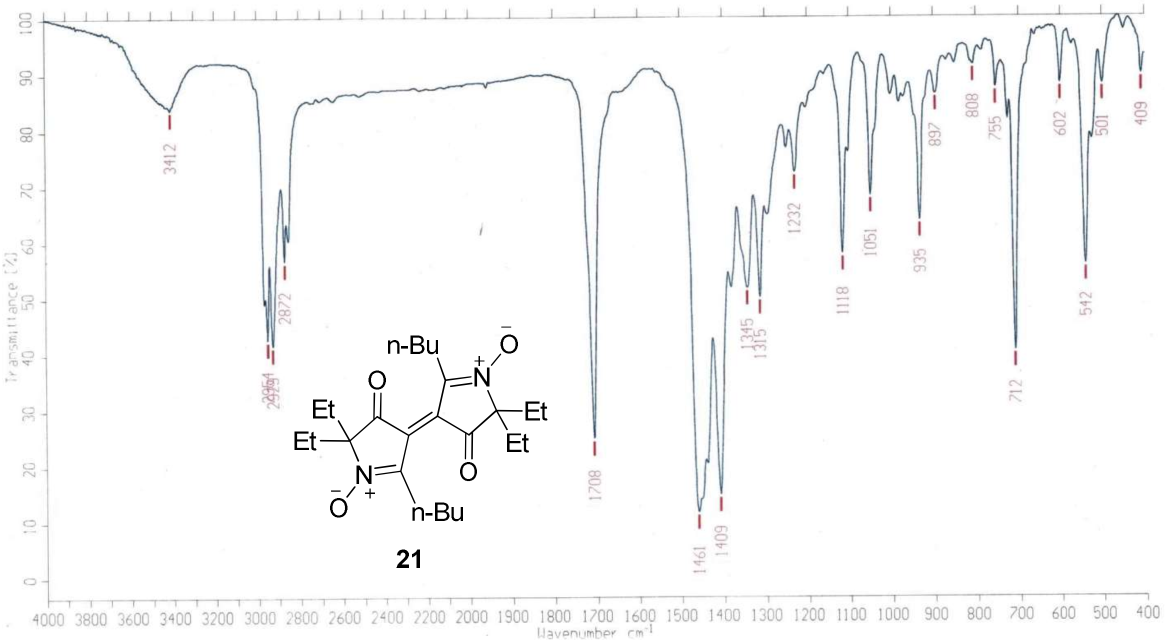

Figure SI11. IR spectrum of **21** (KBr).

1.12 5-(*tert*-Butyl)-5-butyl-2,2-diethyl-3-hydroxypyrrolidin-1-oxyl (**4**)

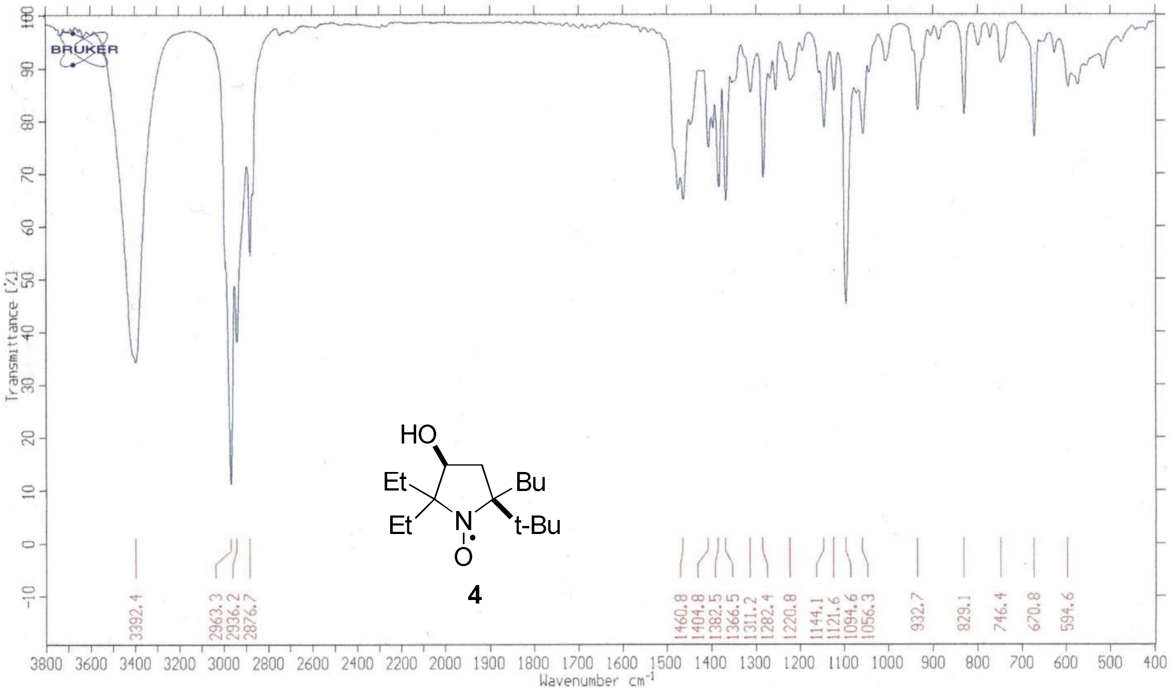

Figure SI12. IR spectrum of **4** (KBr).

1.13 5-(*tert*-Butyl)-2,2-diethyl-3,4-bis(methoxycarbonyl)pyrrolidine (**23a,b**).

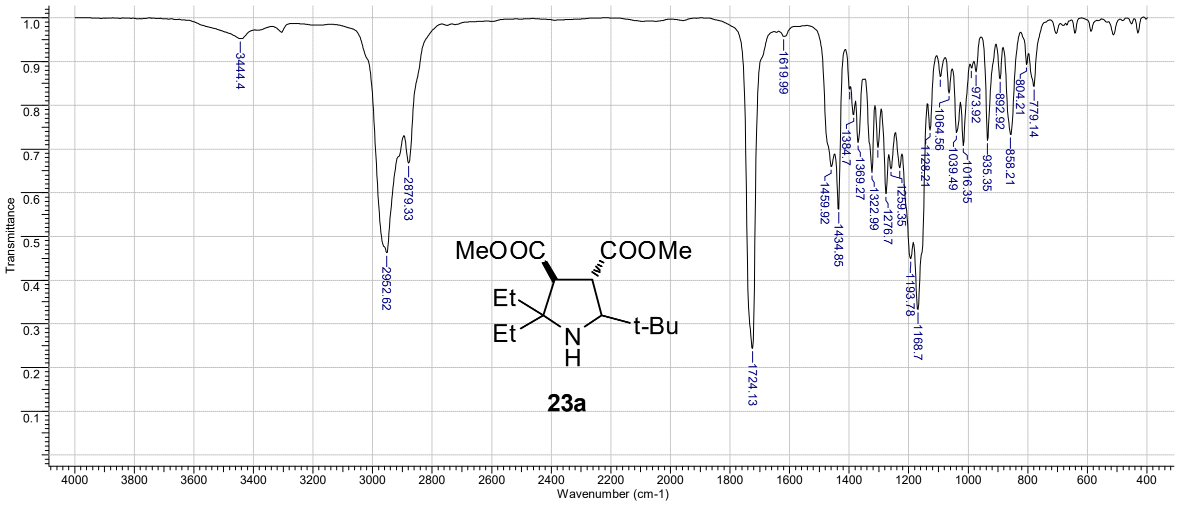

Figure SI13. IR spectrum of **23a** (KBr).

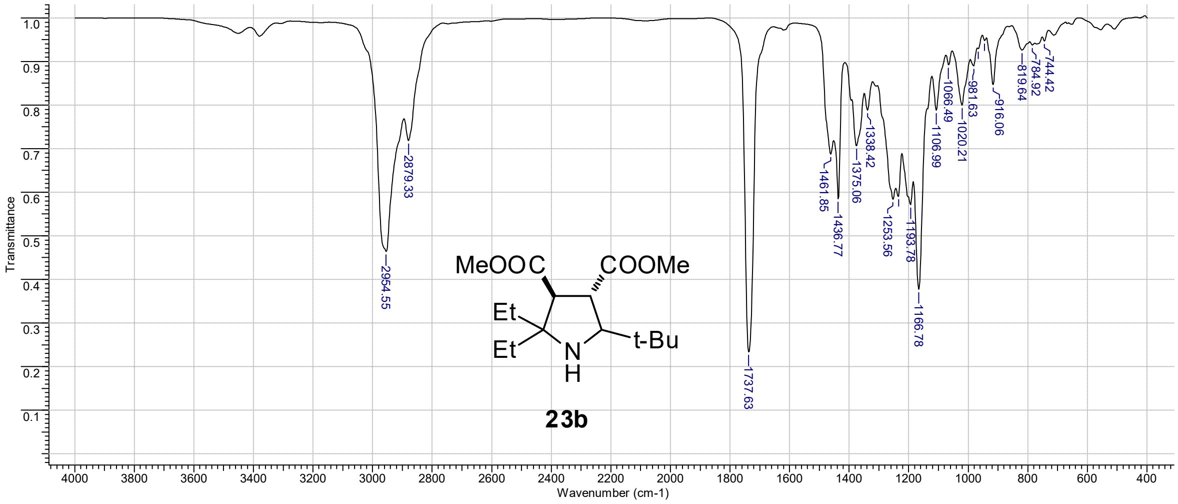

Figure SI14. IR spectrum of **23b** (neat).

1.14 5-(*tert*-Butyl)-2,2-diethyl-3,4-bis(hydroxymethyl)-3,4-dihydro-2*H*-pyrrole 1-oxide (**24**).

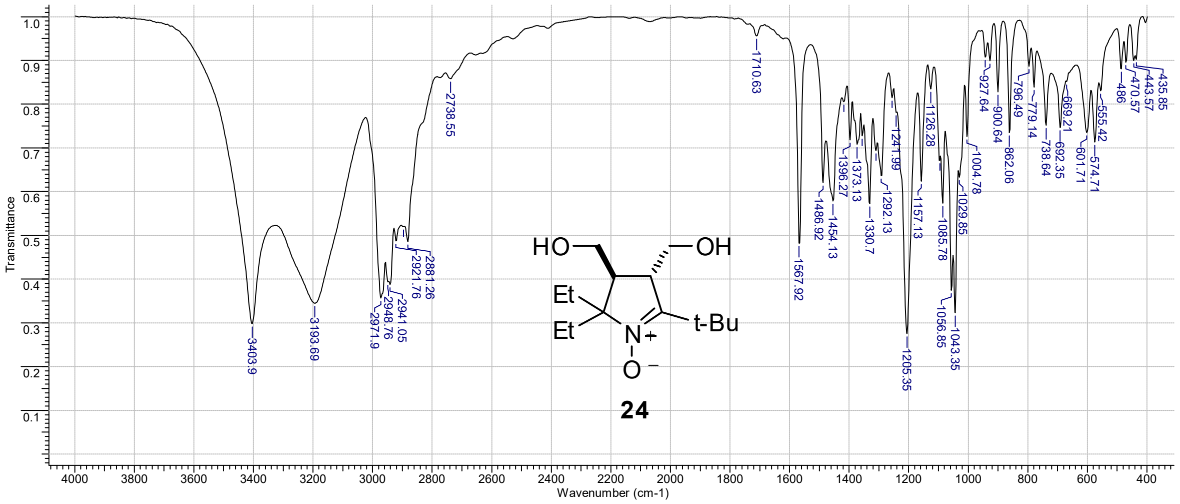

Figure SI15. IR spectrum of **24** (KBr).

1.15 2-(*tert*-Butyl)-2-butyl-5,5-diethyl-3,4-bis(hydroxymethyl)pyrrolidin-1-oxyl (**5**)

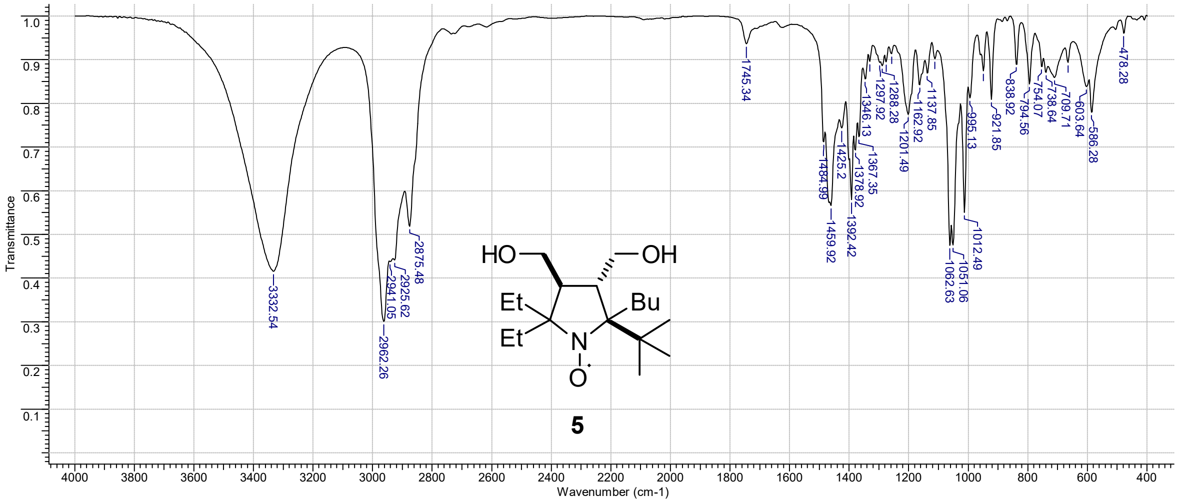

Figure SI16. IR spectrum of **5** (KBr).

1.16 5-(*tert*-Butyl)-2,2-diethyl-3-(hydroxymethyl)-4-pentyl-3,4-dihydro-2*H*-pyrrole (**26**)

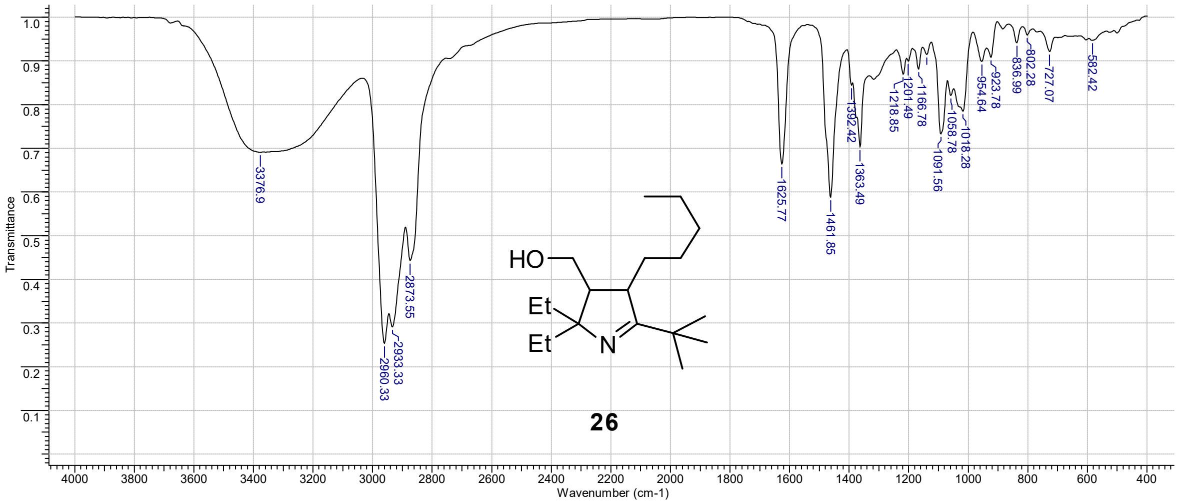

Figure SI17. IR spectrum of **26** (neat).

1.17 3-Carboxy-2,2,5,5-tetraethylpyrrolidin-1-oxyl (**1**) and its' sodium salt (**1Na**).

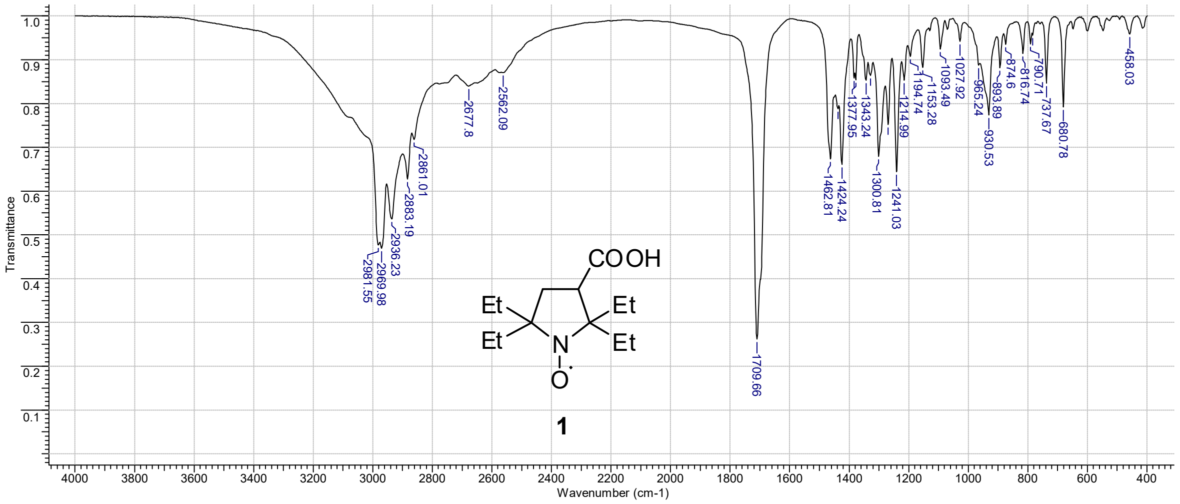

Figure SI18. IR spectrum of **1** (KBr).

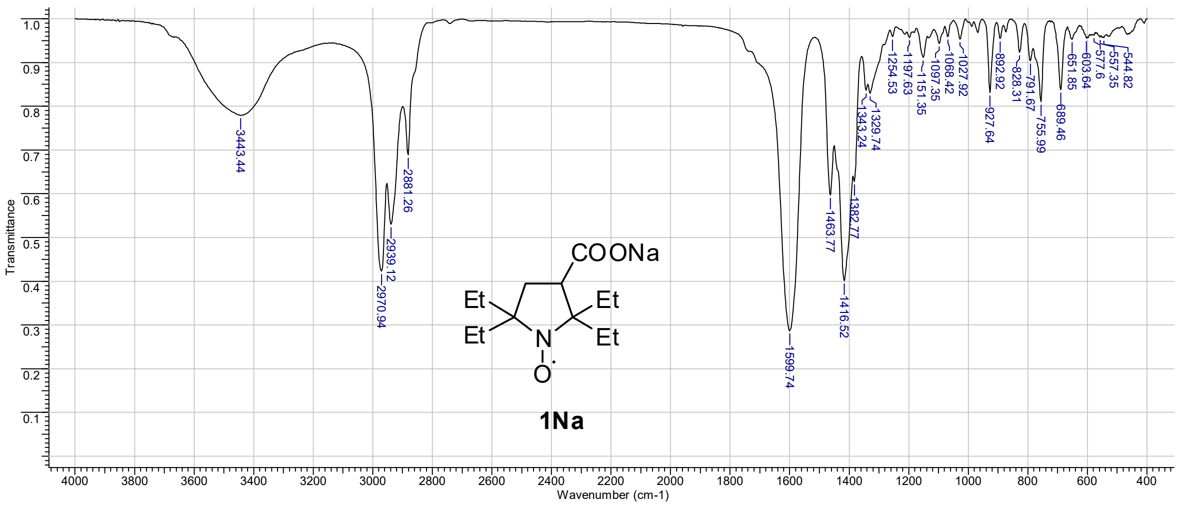

Figure SI19. IR spectrum of **1Na** (KBr).

1.18 2,2,5,5-Tetraethyl-3,4-bis(hydroxymethyl)pyrrolidin-1-oxyl (2)

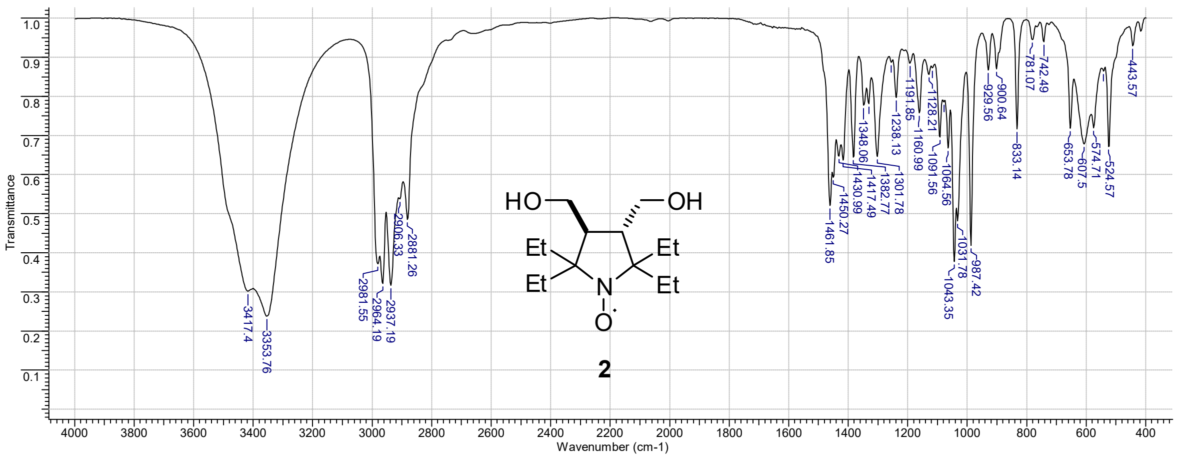

Figure SI20. IR spectrum of **2** (KBr).

2. UV spectral data

2.1 5,5-Diethyl-1-hydroxy-2,2,4-trimethyl-2,5-dihydro-1*H*-imidazol (**7**) (**Fig. SI21**).

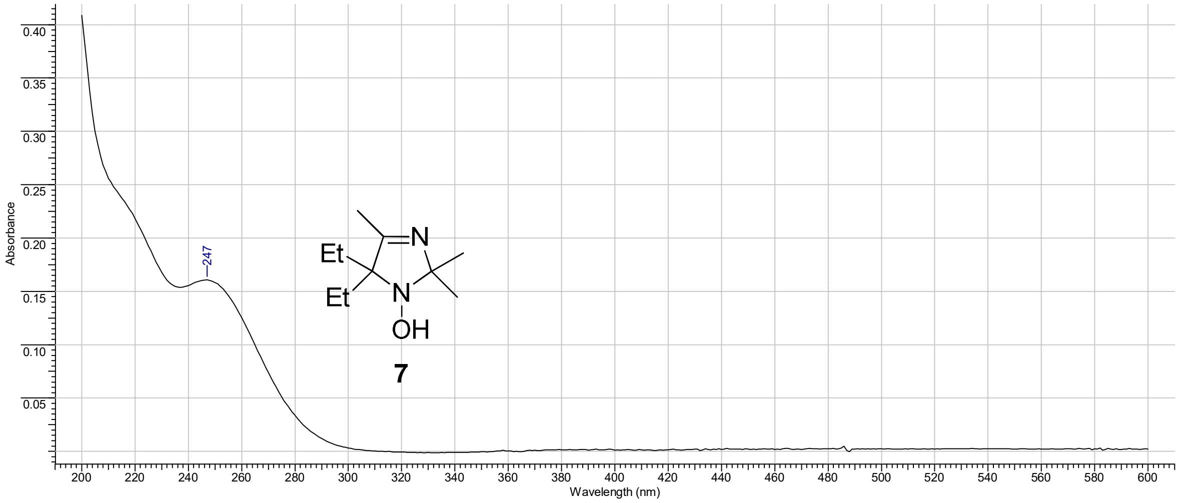

Figure SI21. UV spectrum of **7** in EtOH (c=3.324mg/25ml, L=0.5cm)

2.2 (Z)-1-(5,5-Diethyl-1-hydroxy-2,2-dimethylimidazolidin-4-ylidene)-3,3-dimethylbutan-2-one (**9**) (**Fig. SI22**).

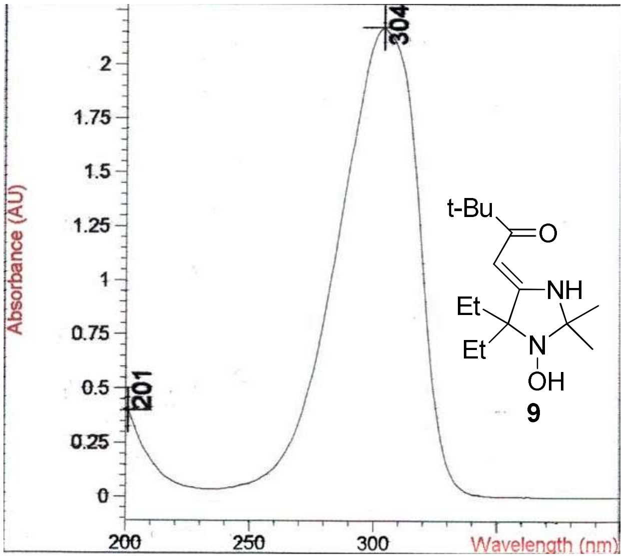

Figure SI22. UV spectrum of **9** in EtOH (c=0.768mg/25ml, L=1.0cm)

2.3 (Z)-4-(3,3-Dimethyl-2-oxobutylidene)-5,5-diethyl-2,2-dimethylimidazolidin-1-oxyl (**10**) (**Fig. SI23**).

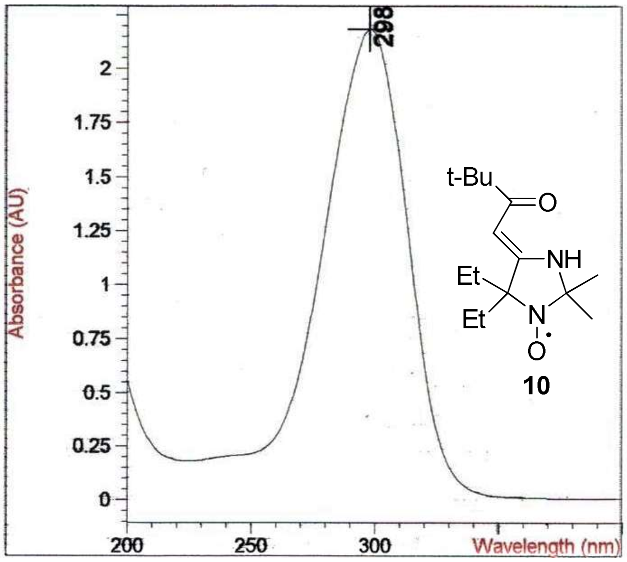

Figure SI23. UV spectrum of **10** in EtOH (c=1.57mg/25ml, L=0.5cm)

2.4 5-(*tert*-Butyl)-2,2-diethyl-3-oxo-3,4-dihydro-2*H*-pyrrole 1-oxide (**6**) (**Fig. SI24**).

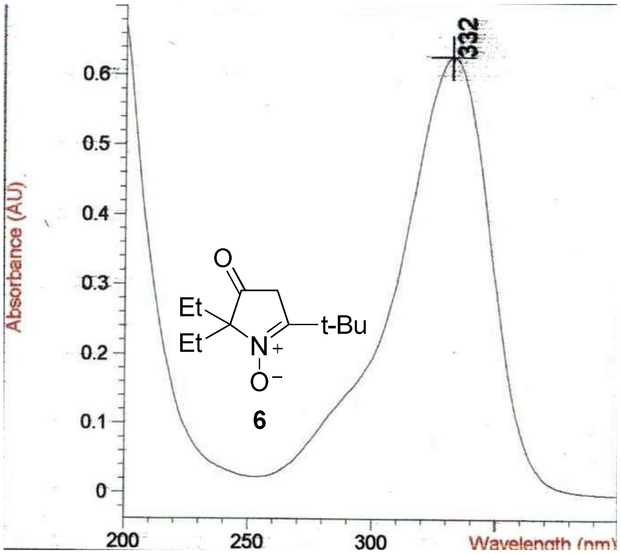

Figure SI24. UV spectrum of **6** in EtOH (c=0.715mg/25ml, L=0.5cm)

2.5 5-(*tert*-Butyl)-2,2-diethyl-3,4-dioxo-3,4-dihydro-2*H*-pyrrole 1-oxide (**17**) (**Fig. SI25**).

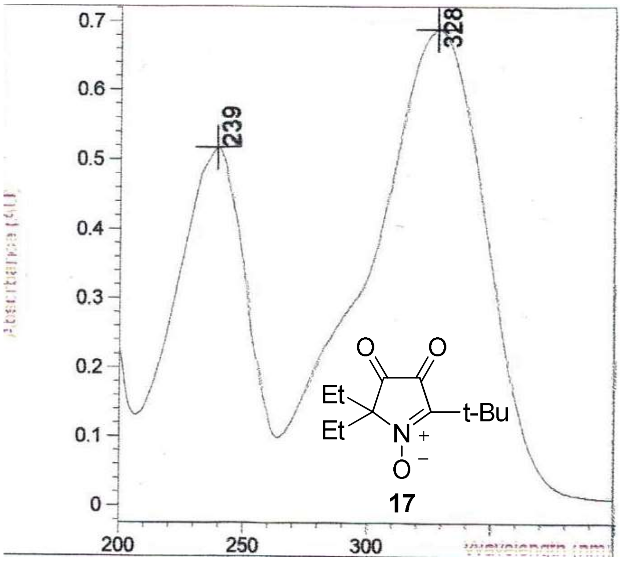

Figure SI25. UV spectrum of **17** in EtOH (c=0.429mg/25ml, L=1.0cm)

2.6 3,3'-bis(2-*tert*-Butyl-5,5-diethyl-4-oxopyrrolinylidene) 1,1'-dioxide (**12**) (**Fig. SI26**).

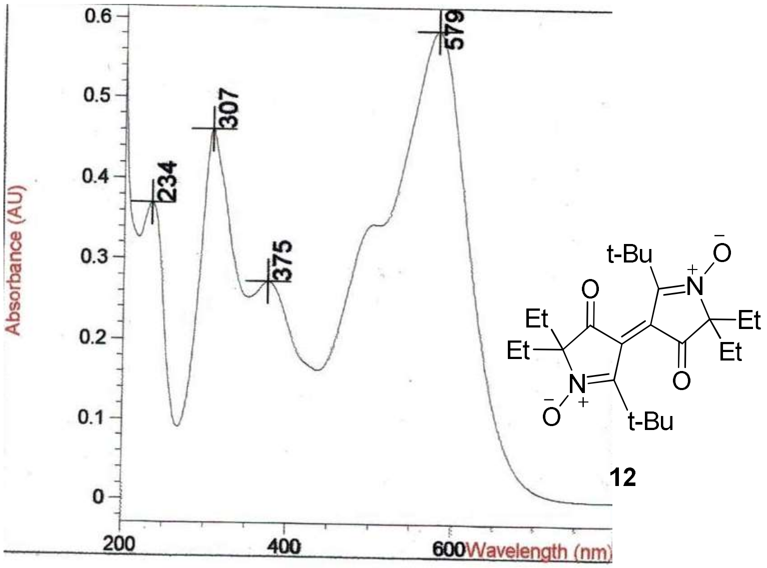

Figure SI26. UV spectrum of **12** in EtOH (c=1.131mg/25ml, L=0.5cm)

2.7 5-(*tert*-Butyl)-5-butyl-2,2-diethyl-3-oxopyrrolidin-1-oxyl (**18**) (**Fig. SI27**).

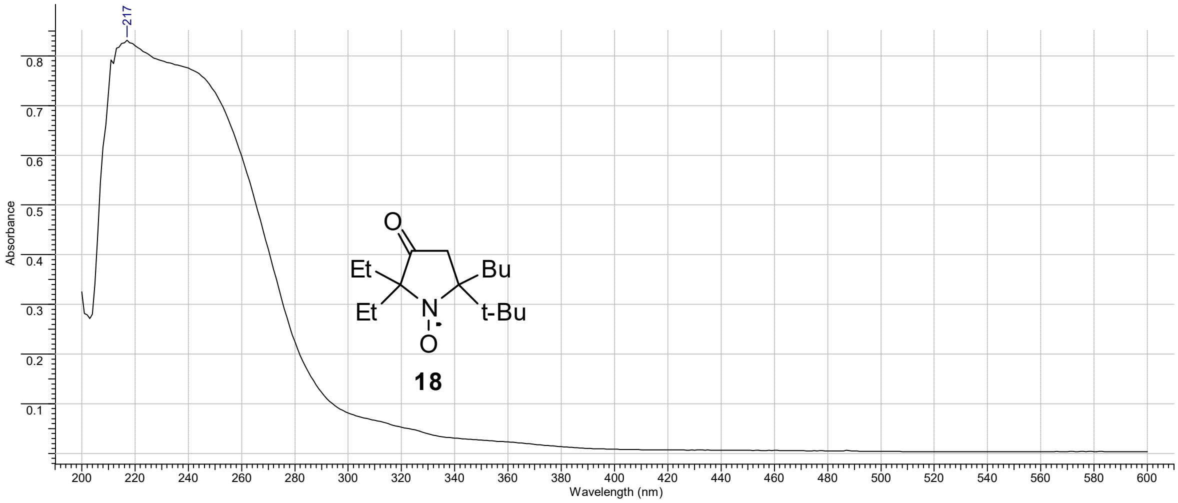

Figure SI27. UV spectrum of **18** in EtOH (c=0,67mg/25ml, L=5.0cm)

2.8 3,3'-bis(2-Butyl-5,5-diethyl-4-oxopyrrolinylidene) 1,1'-dioxide (**21**) (**Fig. SI28**).

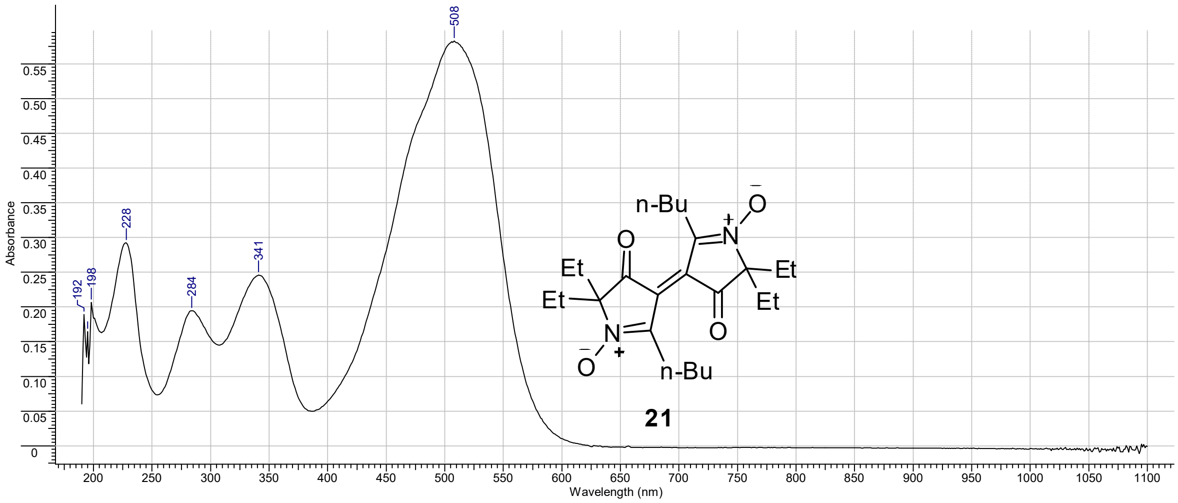

Figure SI28. UV spectrum of **21** in EtOH (c=0.397mg/25ml, L=1 cm)

2.9 5-(*tert*-Butyl)-5-butyl-2,2-diethyl-3-hydroxypyrrolidin-1-oxyl (**4**) (**Fig. SI29**).

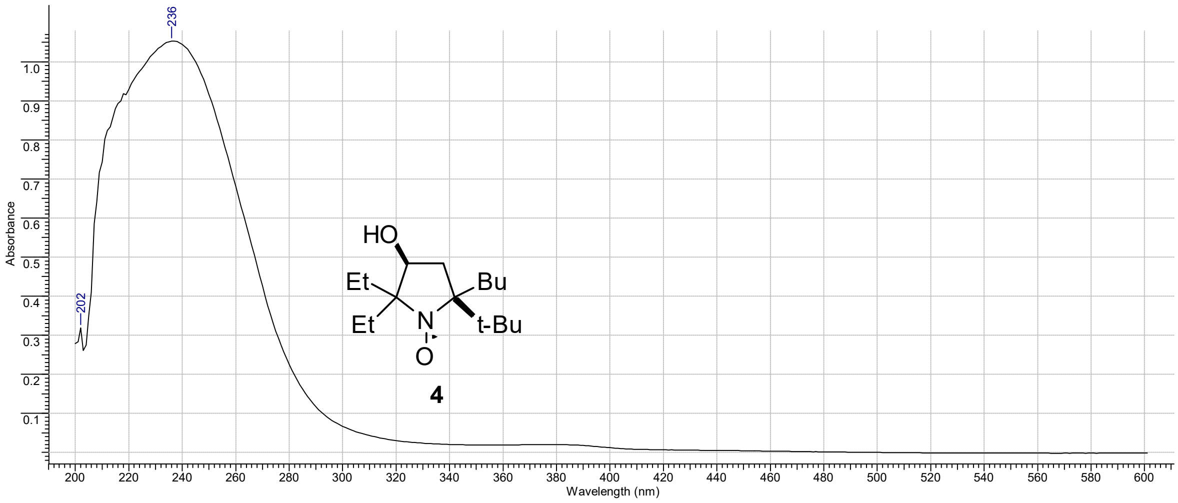

Figure SI29. UV spectrum of **4** in EtOH (c=0,675mg/25ml, L=5cm)

2.10 5-(*tert*-Butyl)-2,2-diethyl-3,4-bis(hydroxymethyl)-3,4-dihydro-2*H*-pyrrole 1-oxide (**24**) (**Fig. SI30**).

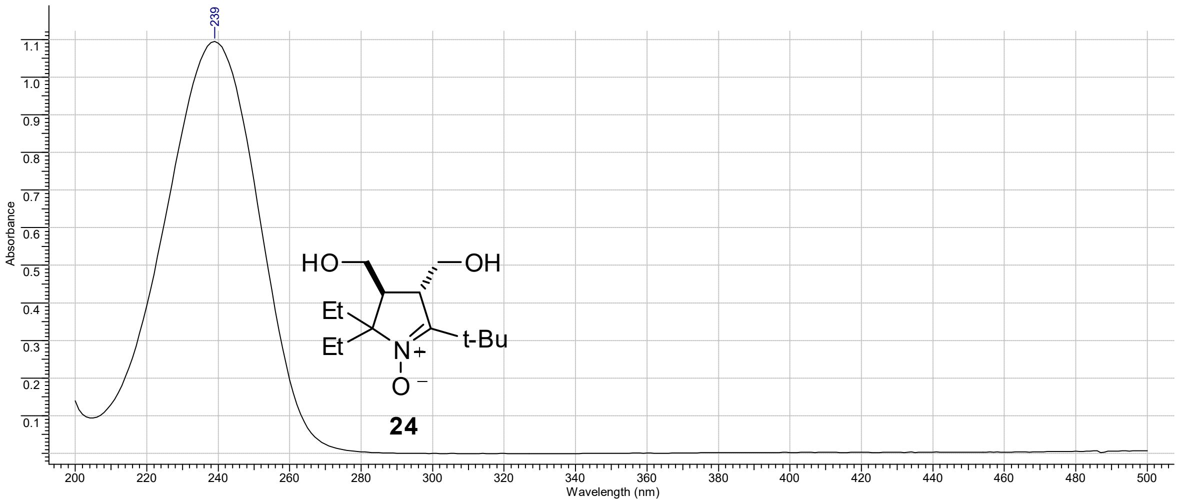

Figure SI30. UV spectrum of **24** in EtOH (c=0.761mg/25ml, L=1cm)

2.11 2-(*tert*-Butyl)-2-butyl-5,5-diethyl-3,4-bis(hydroxymethyl)pyrrolidin-1-oxyl (**5**) (**Fig. SI31**).

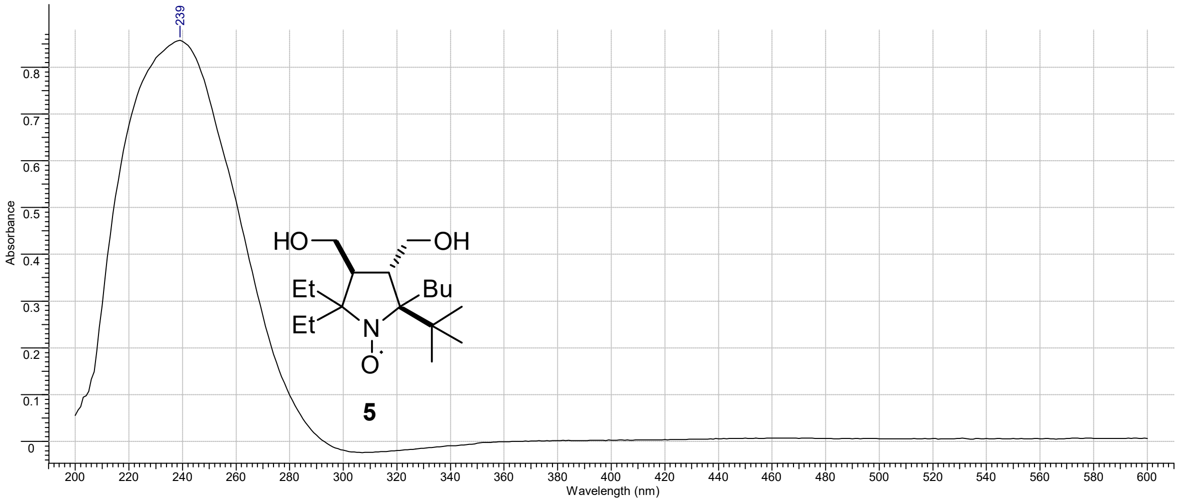

Figure SI31. UV spectrum of **5** in EtOH (c=0,788mg/25ml, L=5cm)

2.12 5-(*tert*-Butyl)-2,2-diethyl-3-(hydroxymethyl)-4-pentyl-3,4-dihydro-2*H*-pyrrole (**26**) (**Fig. SI32**).

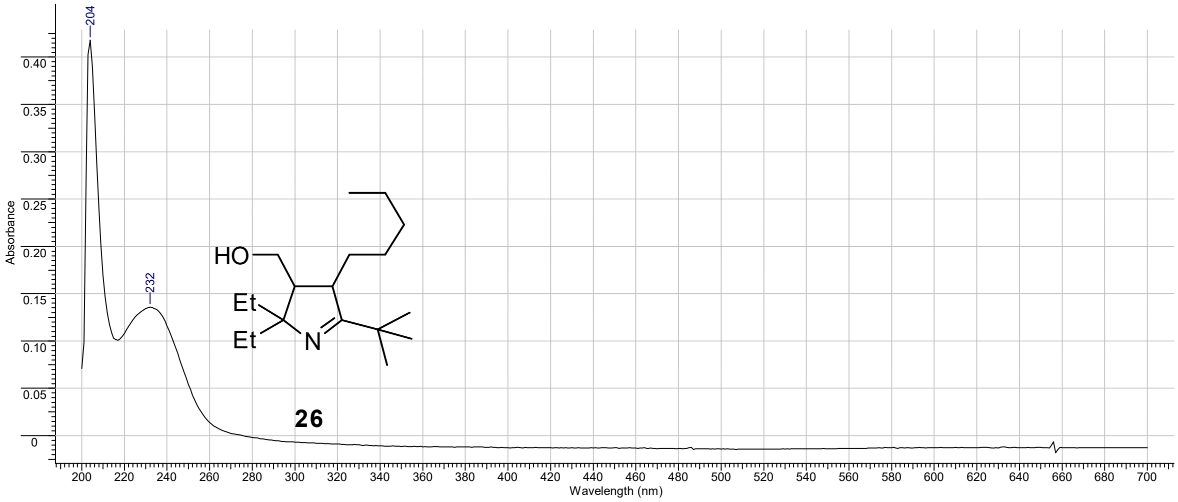

Figure SI32. UV spectrum of **26** in EtOH, (c=1.435mg/25m, L=2cm)

3. ^{1}H and ^{13}C NMR spectral data

3.1 5,5-Diethyl-1-hydroxy-2,2,4-trimethyl-2,5-dihydro-1*H*-imidazol (**7**)

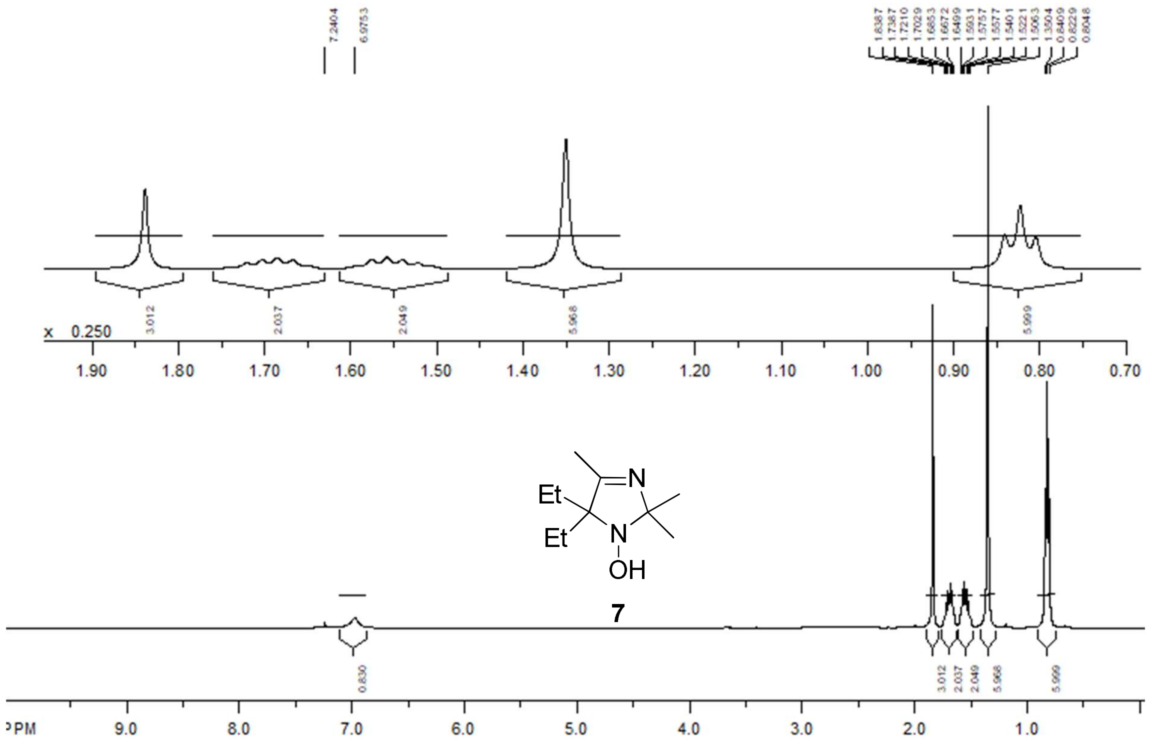

Figure SI33. ^{1}H NMR spectrum of **7** in $CDCl_3$ at 400 MHz

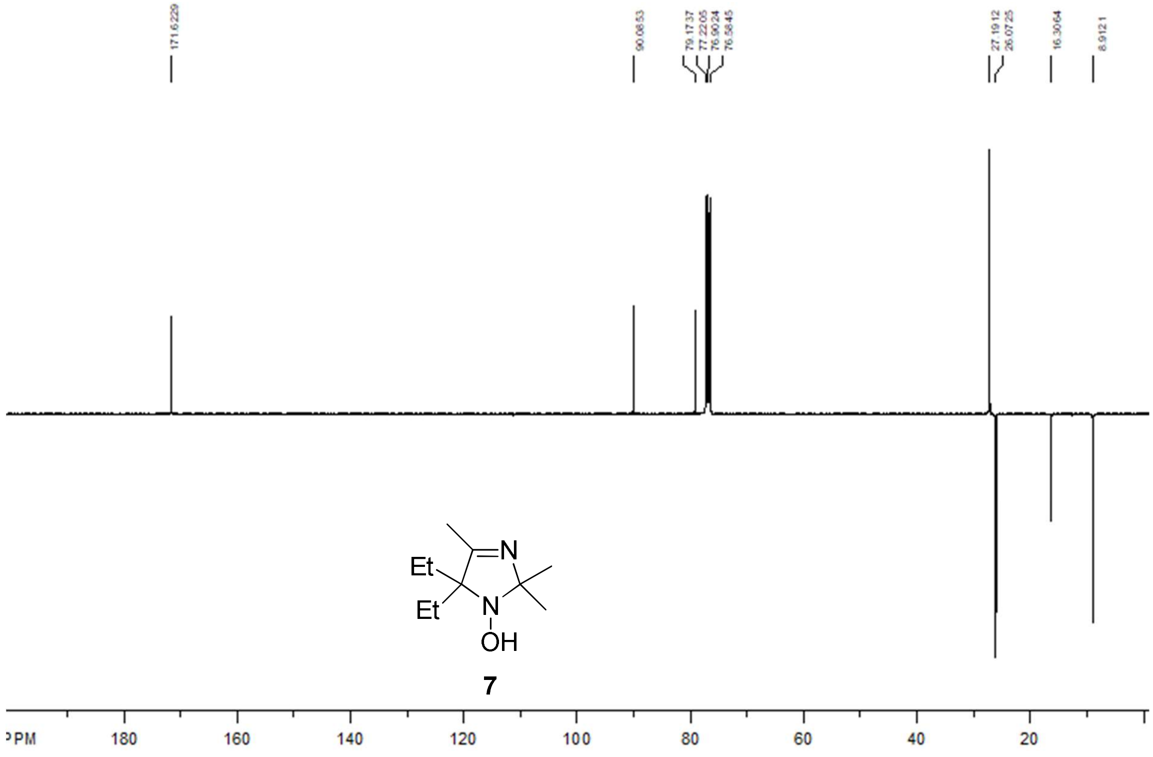

Figure SI34. ^{13}C NMR spectrum of **7** in $CDCl_3$ at 100 MHz

3.2 (Z)-1-(5,5-Diethyl-1-hydroxy-2,2-dimethylimidazolidin-4-ylidene)-3,3-dimethylbutan-2-one (**9**)

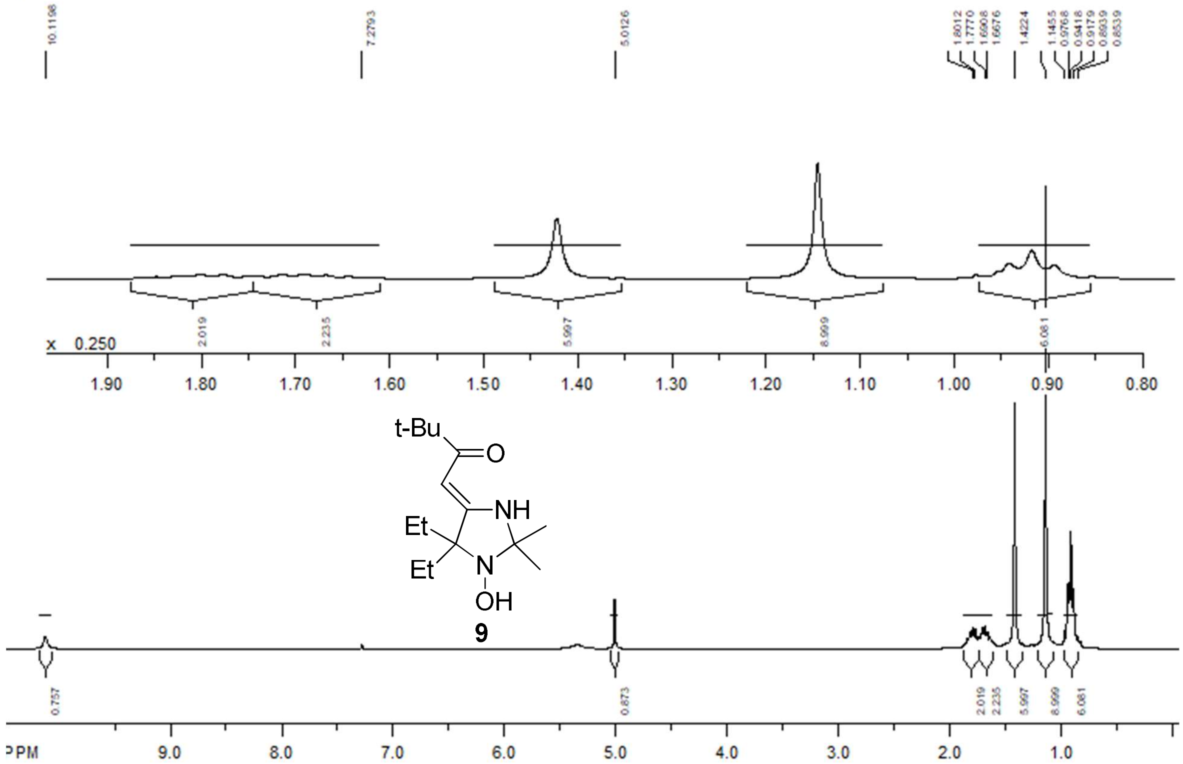

Figure SI35. ^{1}H NMR spectrum of **9** in $CDCl_3$ at 300 MHz

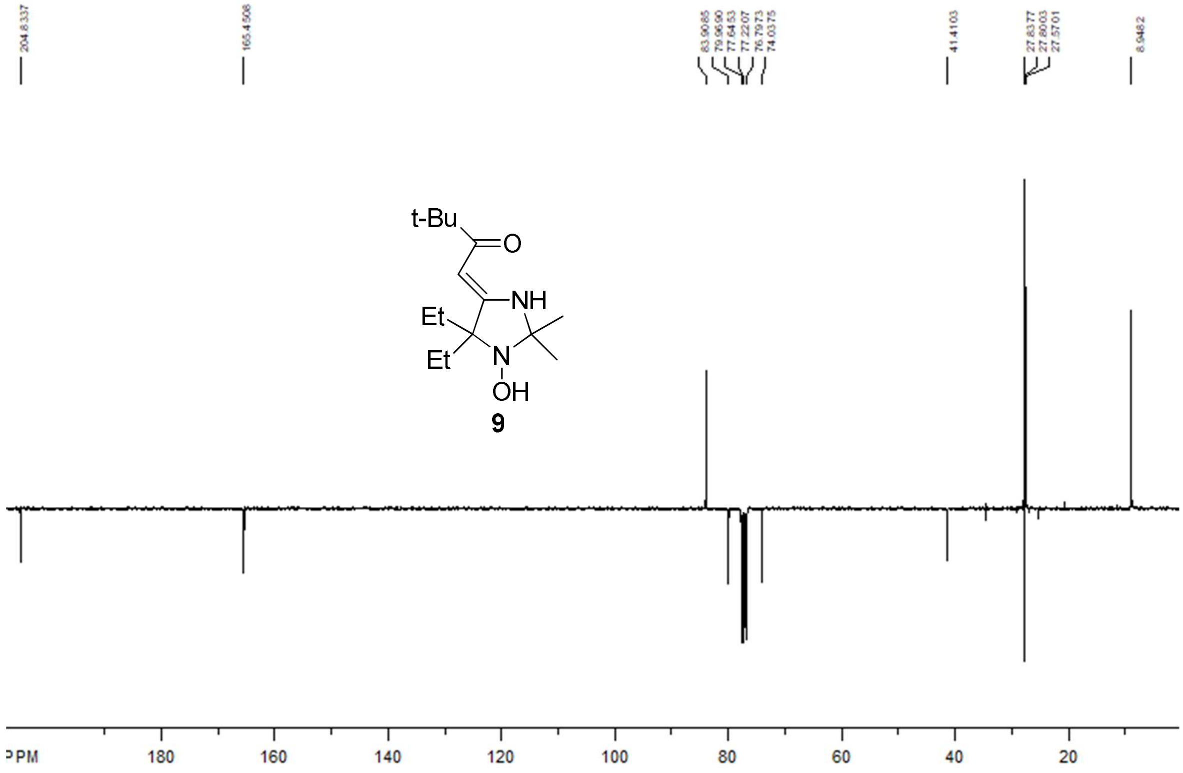

Figure SI36. ^{13}C NMR spectrum of **9** in $CDCl_3$ at 75 MHz

3.3 5-(*tert*-Butyl)-2,2-diethyl-3-oxo-3,4-dihydro-2*H*-pyrrole 1-oxide (**6**)

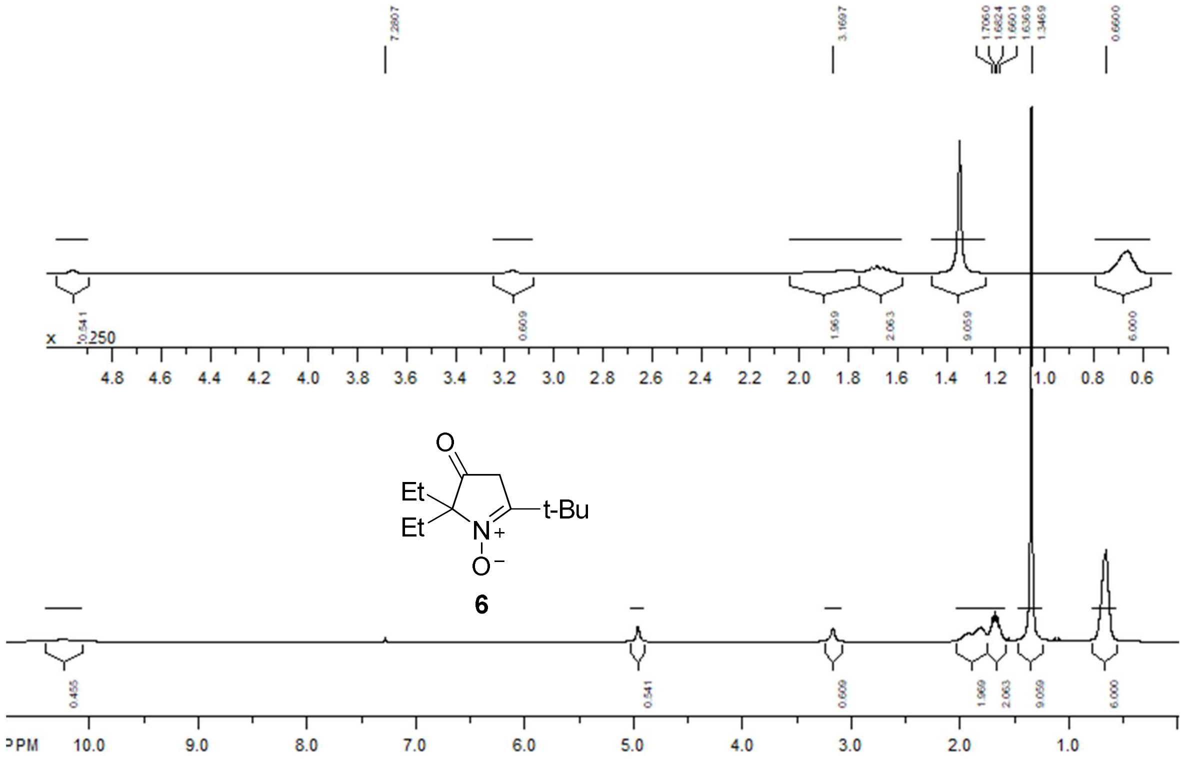

Figure SI37. ^{1}H NMR spectrum of **6** in $CDCl_3$ at 300 MHz

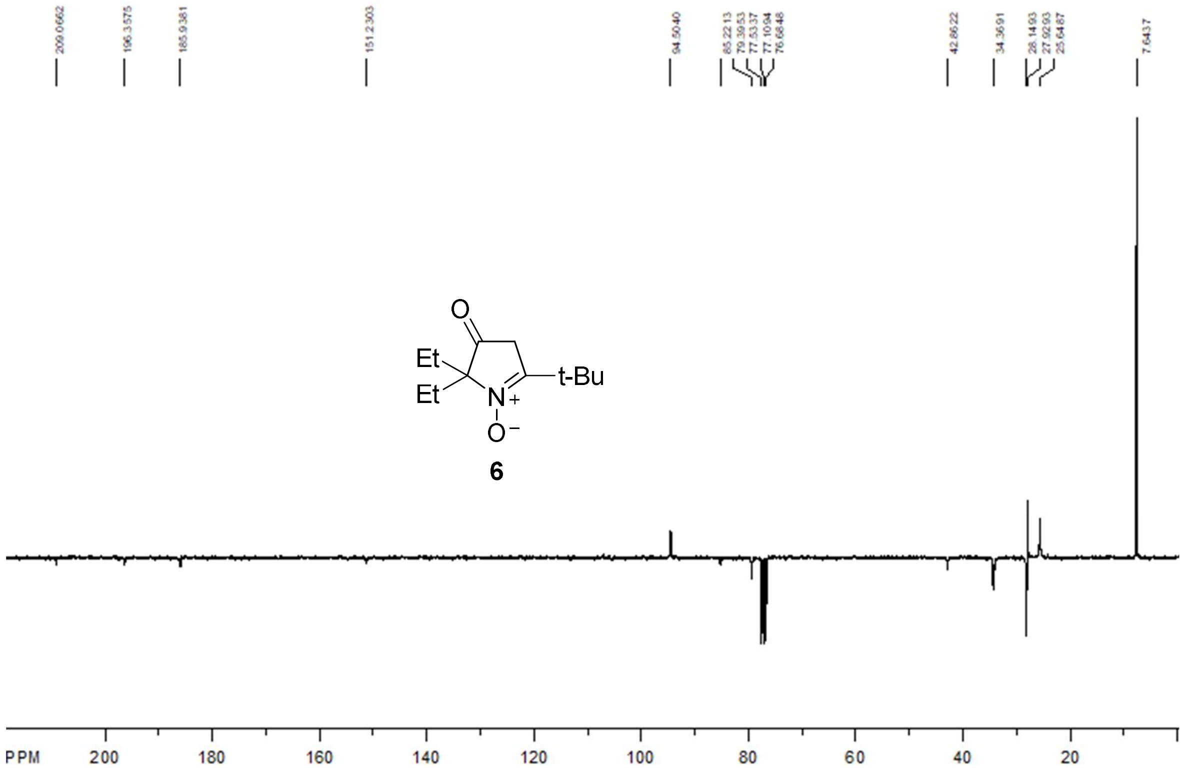

Figure SI38. ^{13}C NMR spectrum of **6** in $CDCl_3$ at 75 MHz

3.4 3-Amino-5-(*tert*-butyl)-2,2-diethyl-2*H*-pyrrole 1-oxide (**13**)

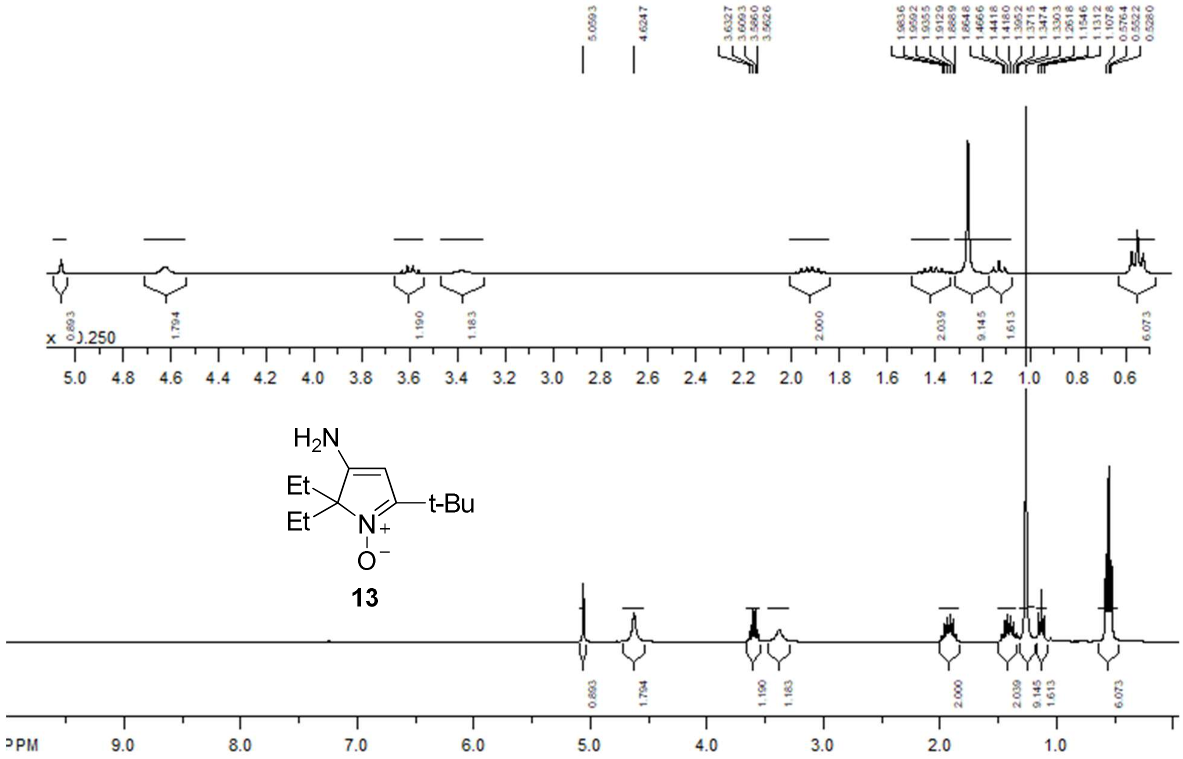

Figure SI39. ^1H NMR spectrum of **13** in $CDCl_3$ at 300 MHz

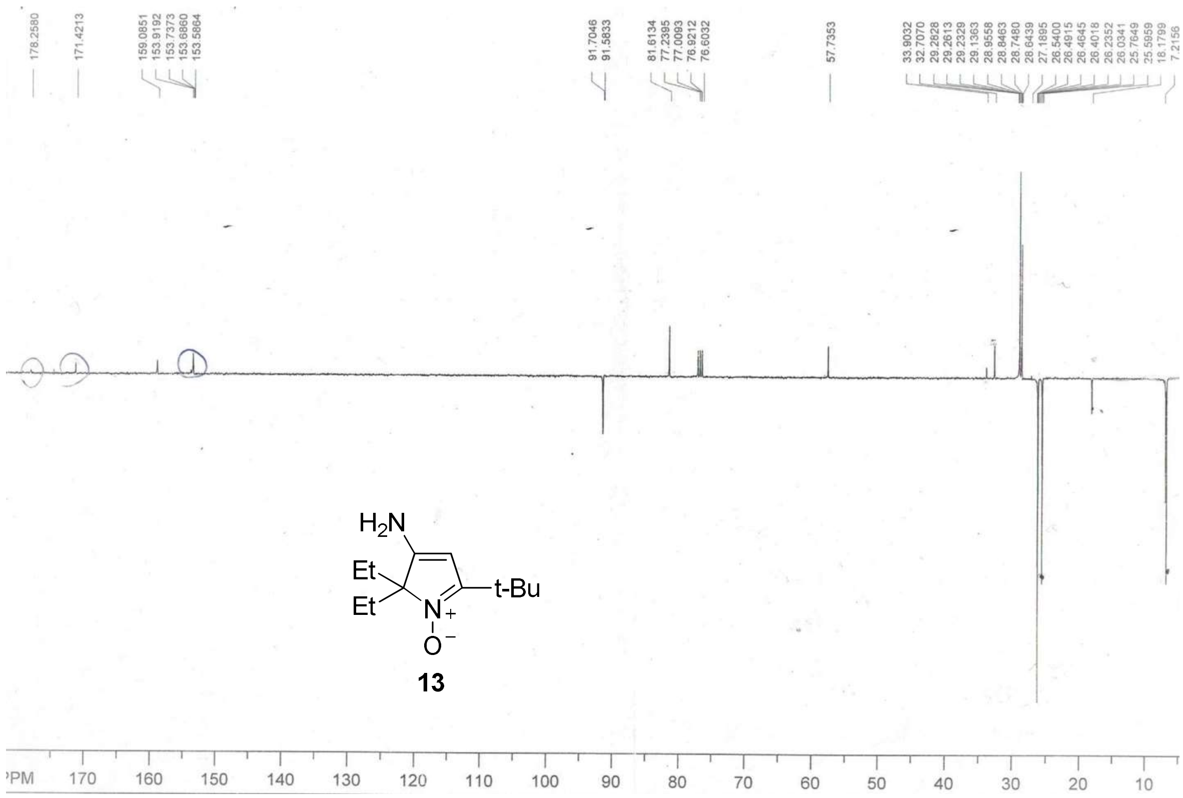

Figure SI40. ^{13}C NMR spectrum of **13** in $CDCl_3$ at 75 MHz

3.5 5-(*tert*-Butyl)-2,2-diethyl-3-imino-4-oxo-3,4-dihydro-2*H*-pyrrole 1-oxide (**16**)

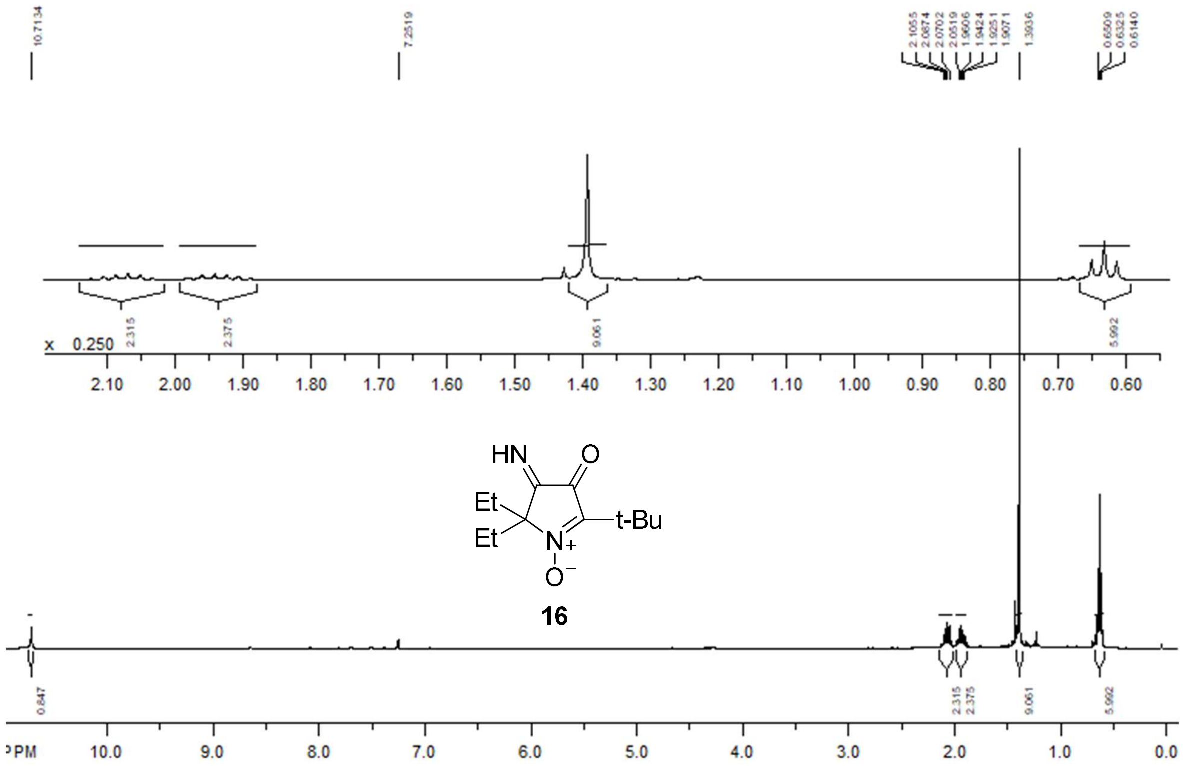

Figure SI41. ^{1}H NMR spectrum of **16** in $CDCl_3$ at 400 MHz

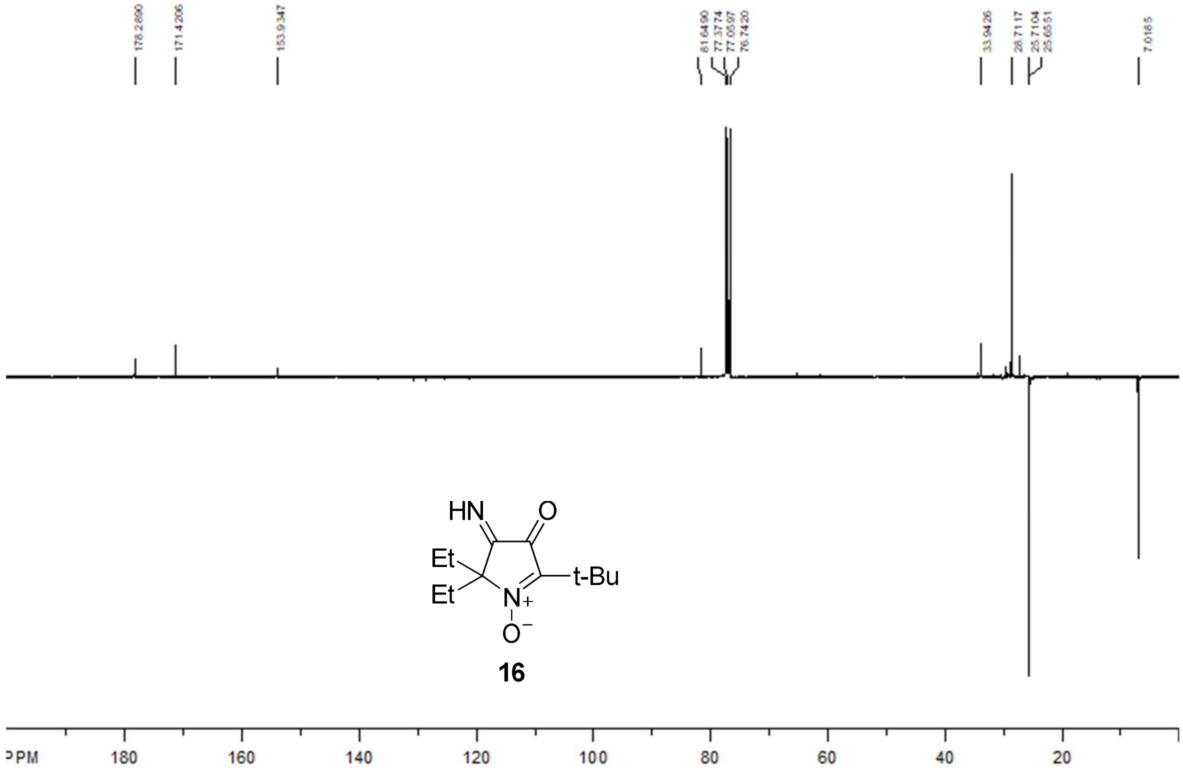

Figure SI42. ^{13}C NMR spectrum of **16** in $CDCl_3$ at 100 MHz

3.6 5-(*tert*-Butyl)-2,2-diethyl-3,4-dioxo-3,4-dihydro-2*H*-pyrrole 1-oxide (**17**)

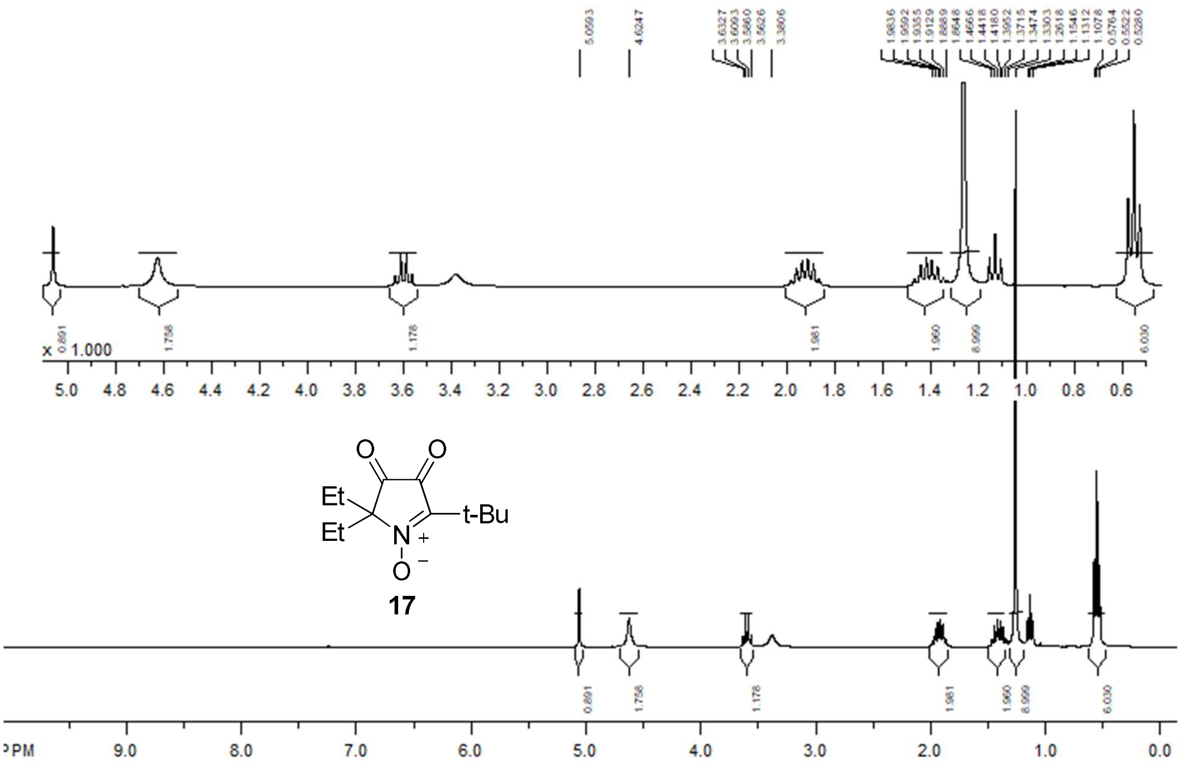

Figure SI43. ^{1}H NMR spectrum of **17** in $CDCl_3$ at 400 MHz

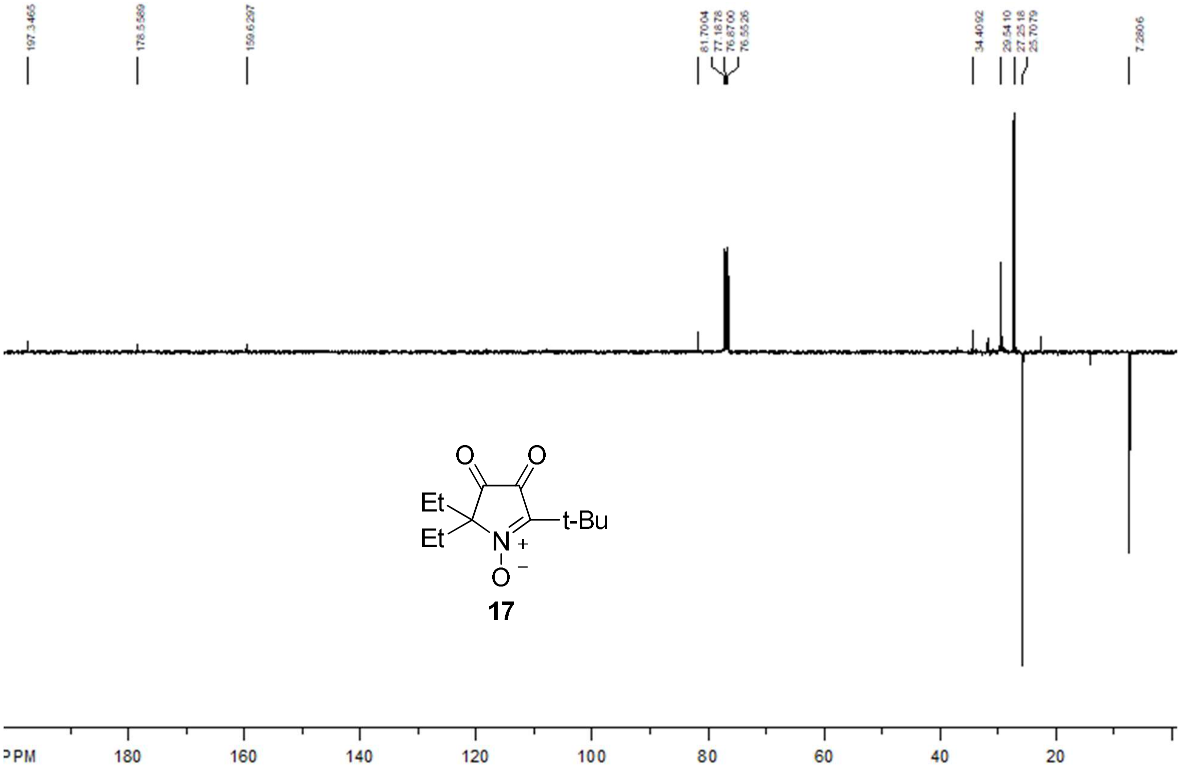

Figure SI44. ^{13}C NMR spectrum of **17** in $CDCl_3$ at 100 MHz

3.7 3,3'-bis(2-*tert*-Butyl-5,5-diethyl-4-oxopyrrolinylidene) 1,1'-dioxide (**12**)

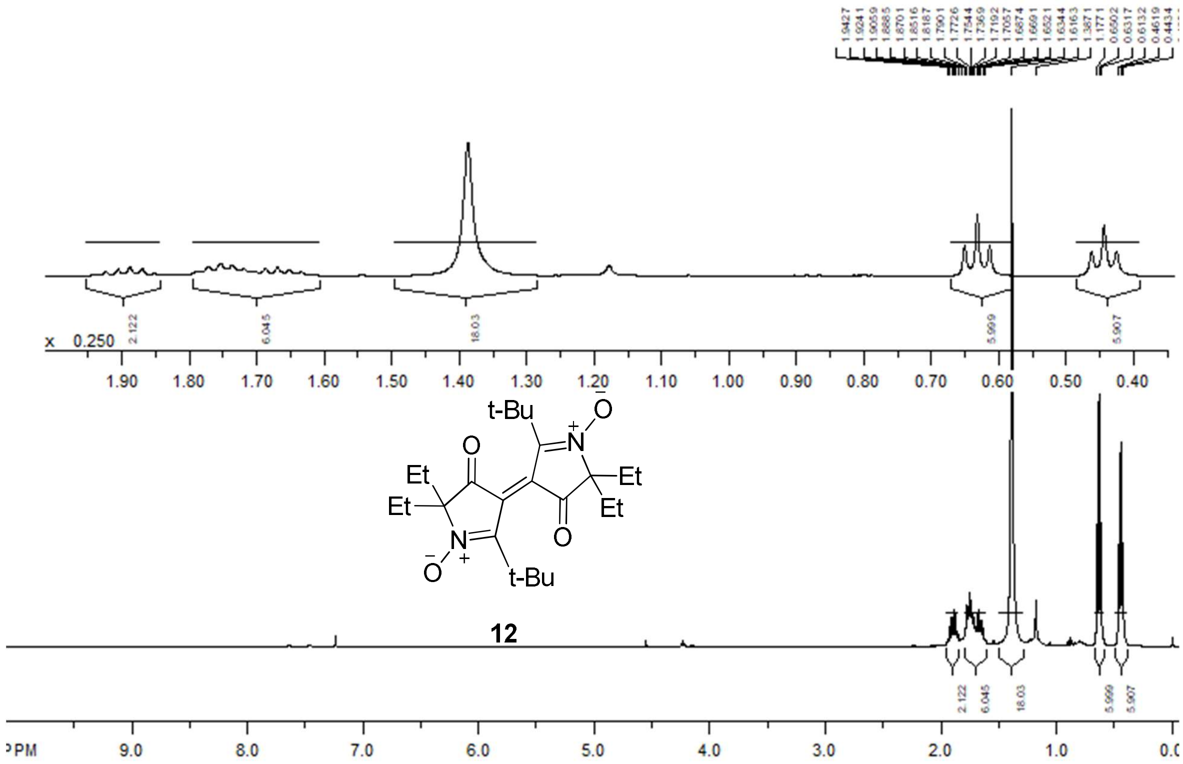

Figure SI45. ^{1}H NMR spectrum of **12** in $CDCl_3$ at 300 MHz

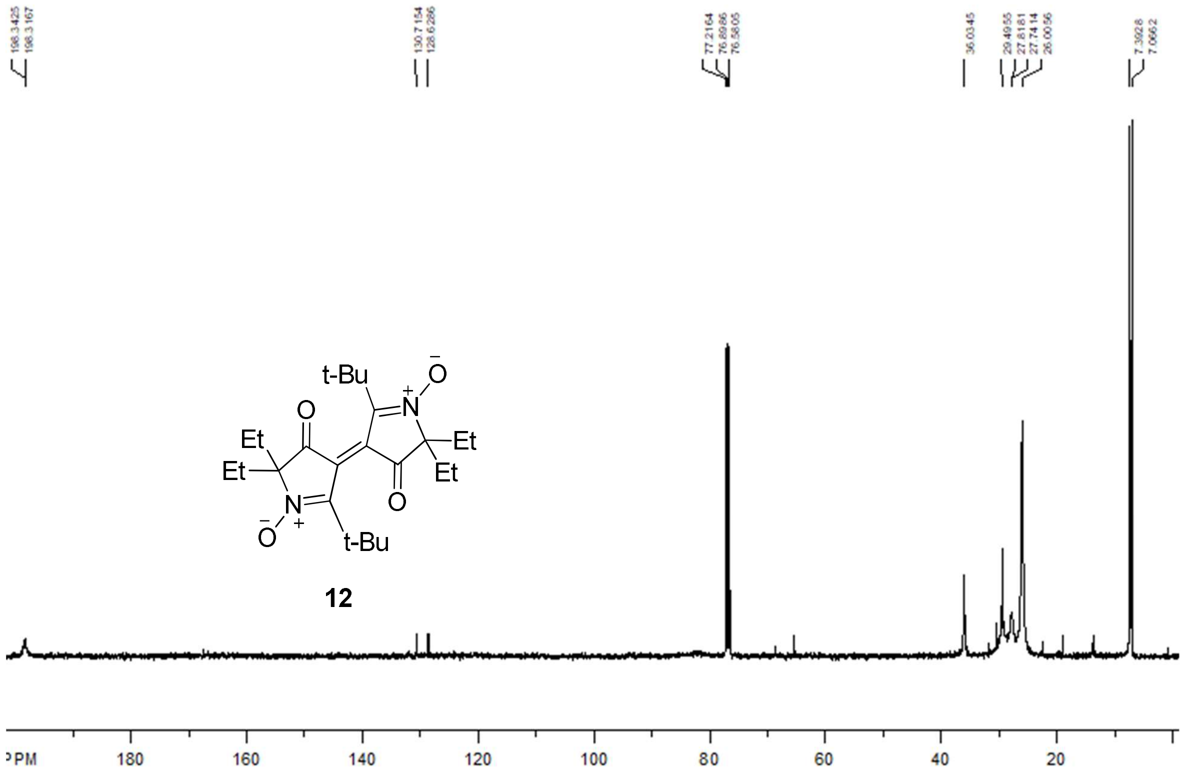

Figure SI46. ^{13}C BB NMR spectrum of **12** in $CDCl_3$ at 75 MHz

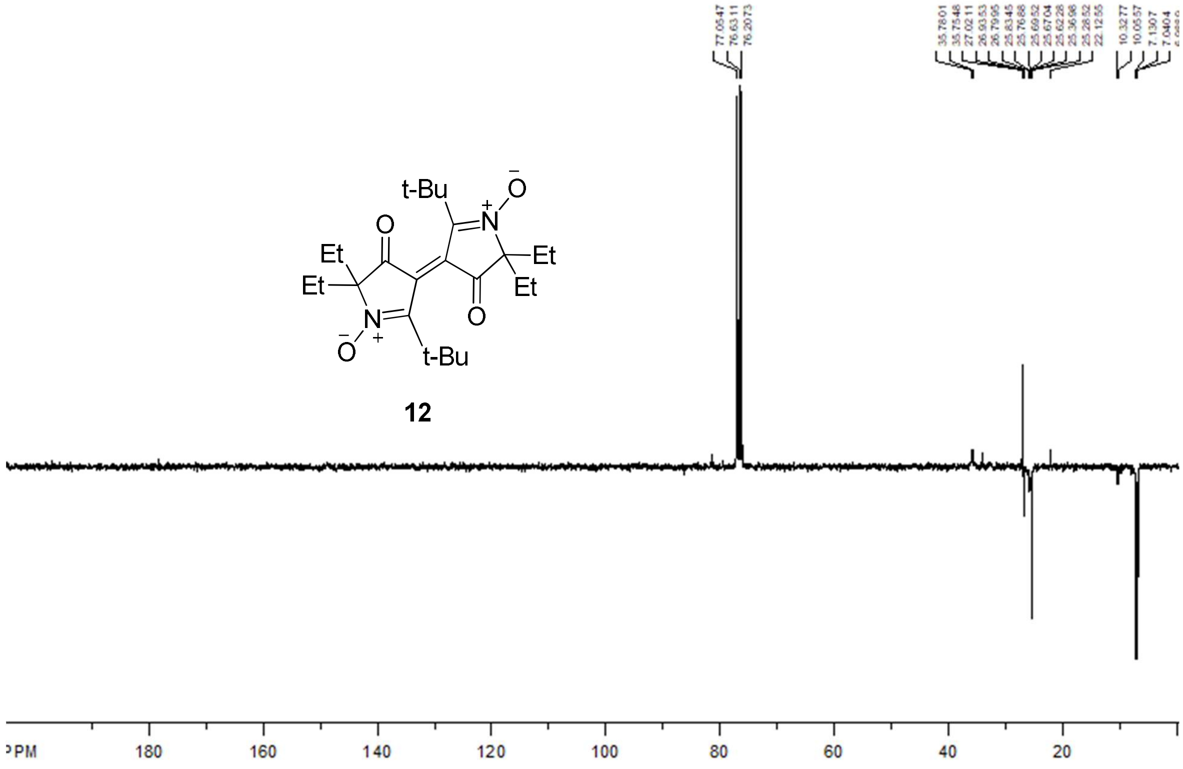

Figure SI47. ^{13}C J-mod NMR spectrum of **12** in $CDCl_3$ at 75 MHz

3.8 1-(*tert*-Butoxy)-2,2,6,6-tetramethylpiperidine (**20**).

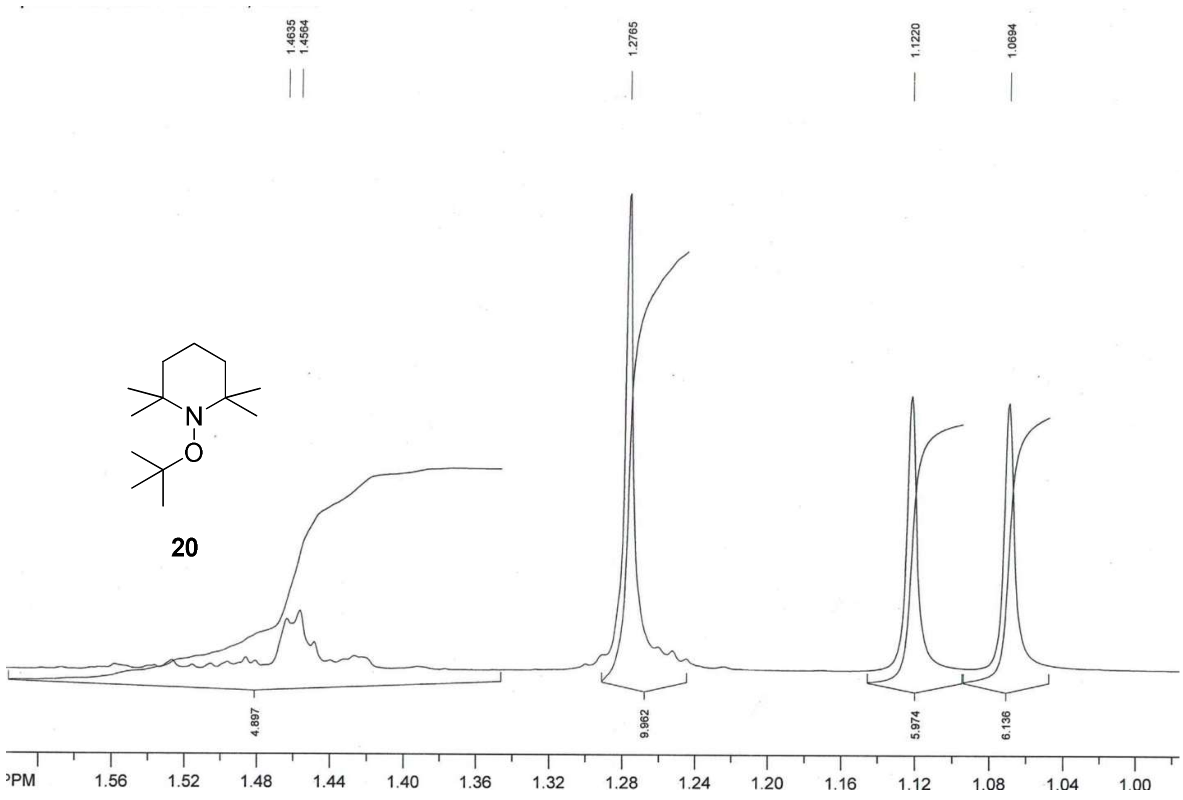

Figure SI48. ^{1}H NMR spectrum of **20** in $CDCl_3$ at 400 MHz

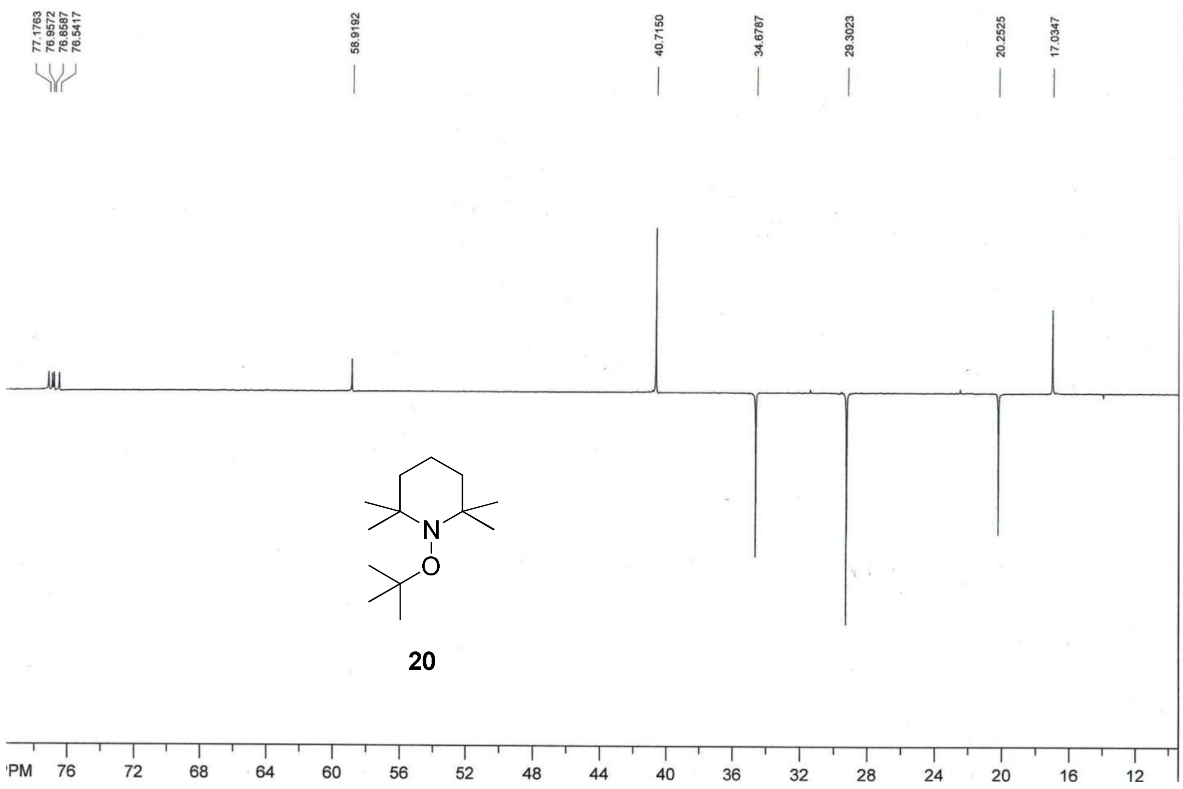

Figure SI49. ^{13}C NMR spectrum of **20** in $CDCl_3$ at 100 MHz

3.9 3,3'-bis(2-Butyl-5,5-diethyl-4-oxopyrrolinylidene) 1,1'-dioxide (**21**)

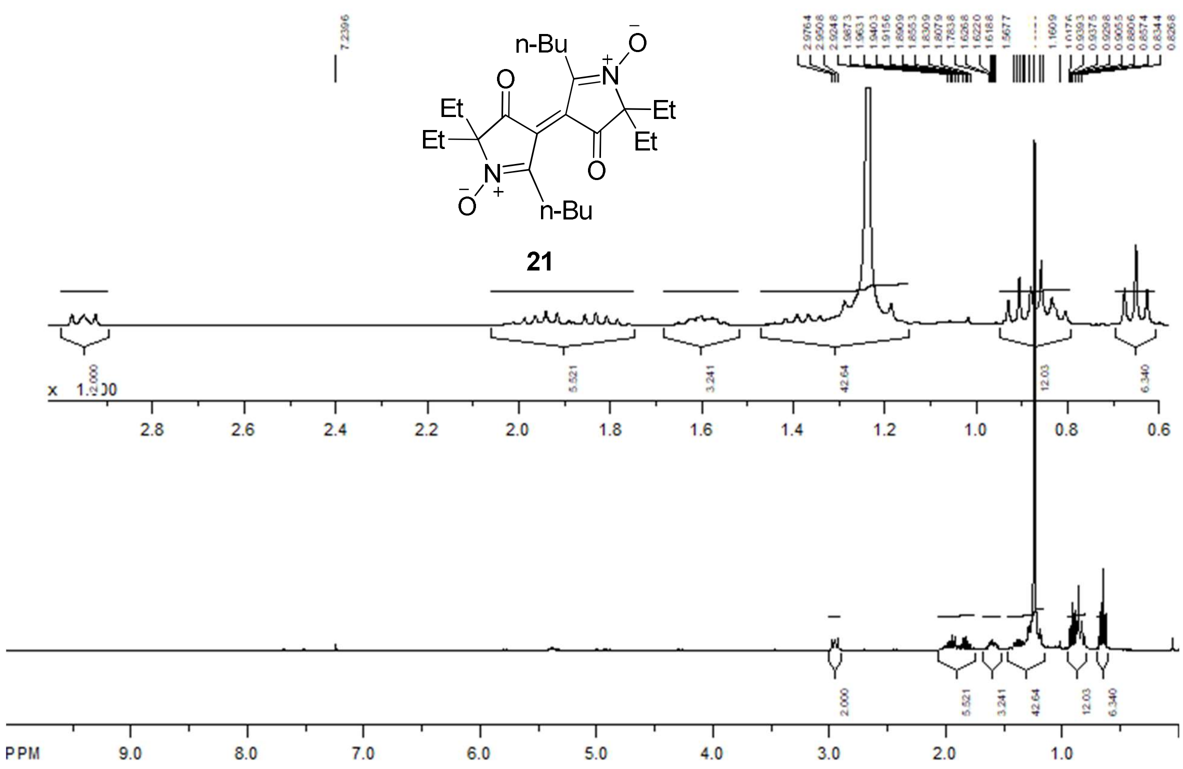

Figure SI50. ^{1}H NMR spectrum of **21** in $CDCl_3$ at 300 MHz

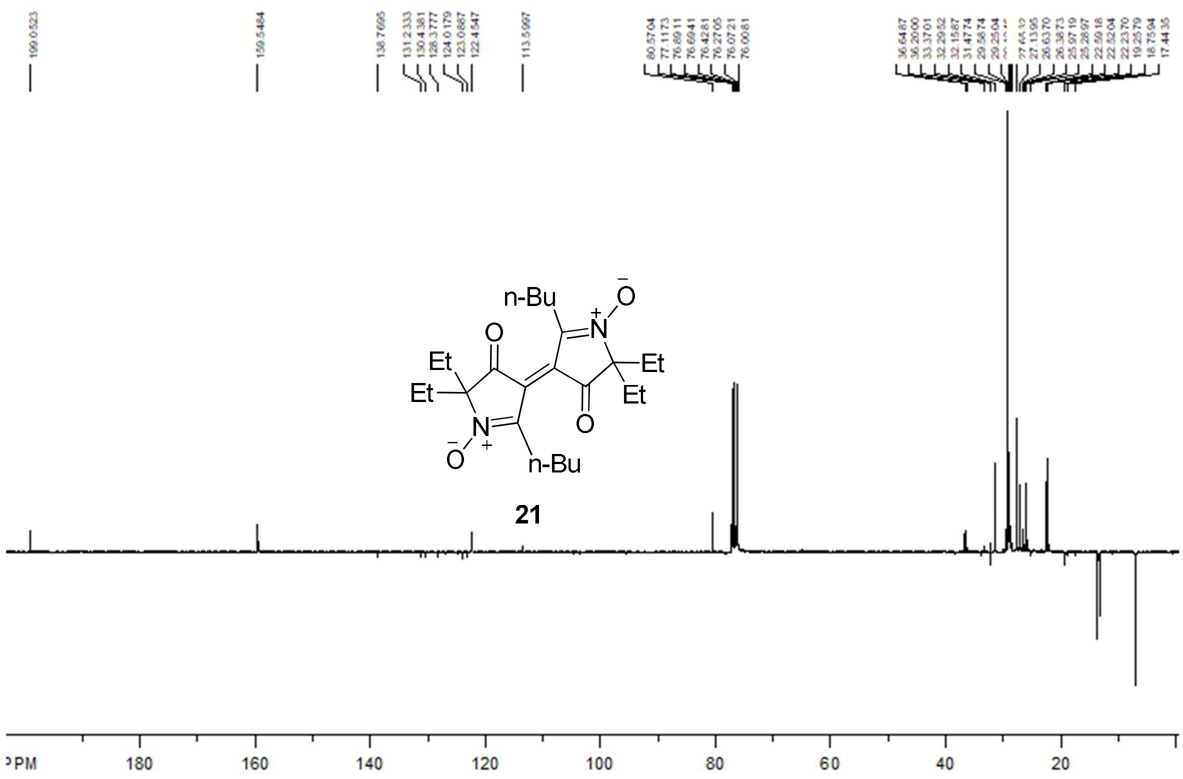

Figure SI51. ^{13}C NMR spectrum of **21** in $CDCl_3$ at 75 MHz

3.10 5-(*tert*-Butyl)-2,2-diethyl-3,4-bis(methoxycarbonyl)pyrrolidine (**23a,b**)

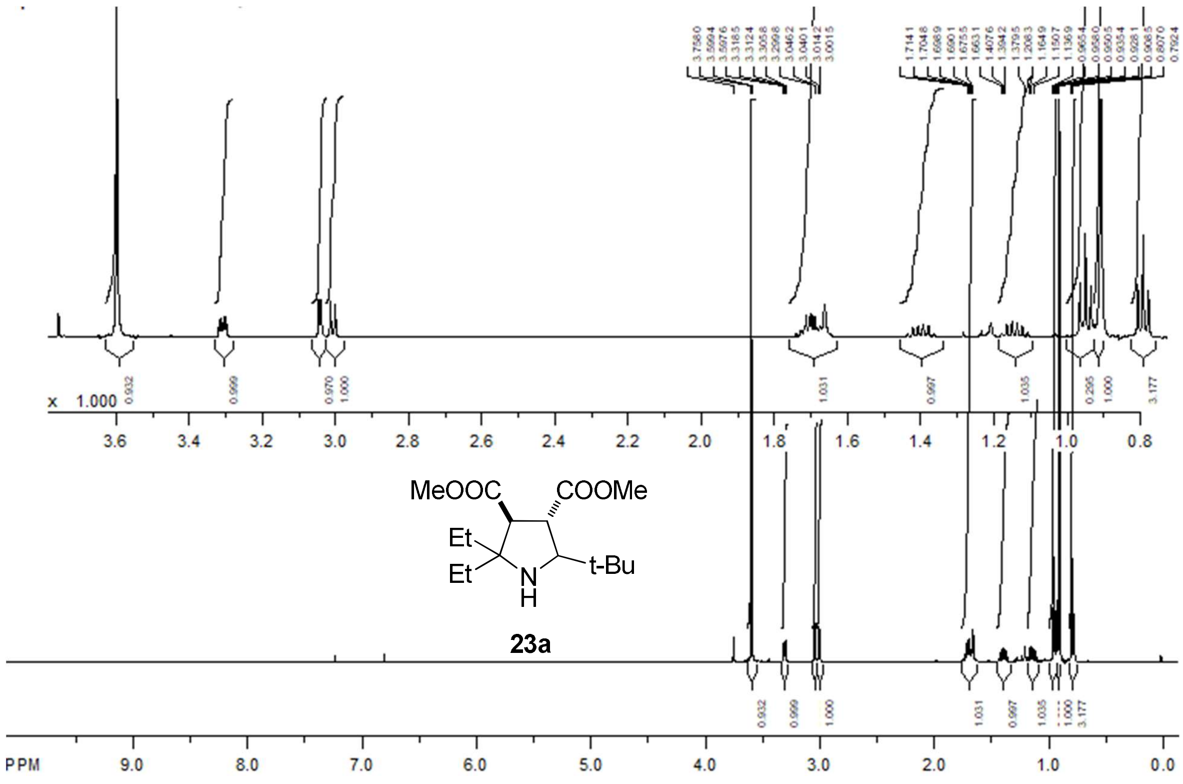

Figure SI52. ^{1}H NMR spectrum of **23a** in $CDCl_3$ at 300 MHz

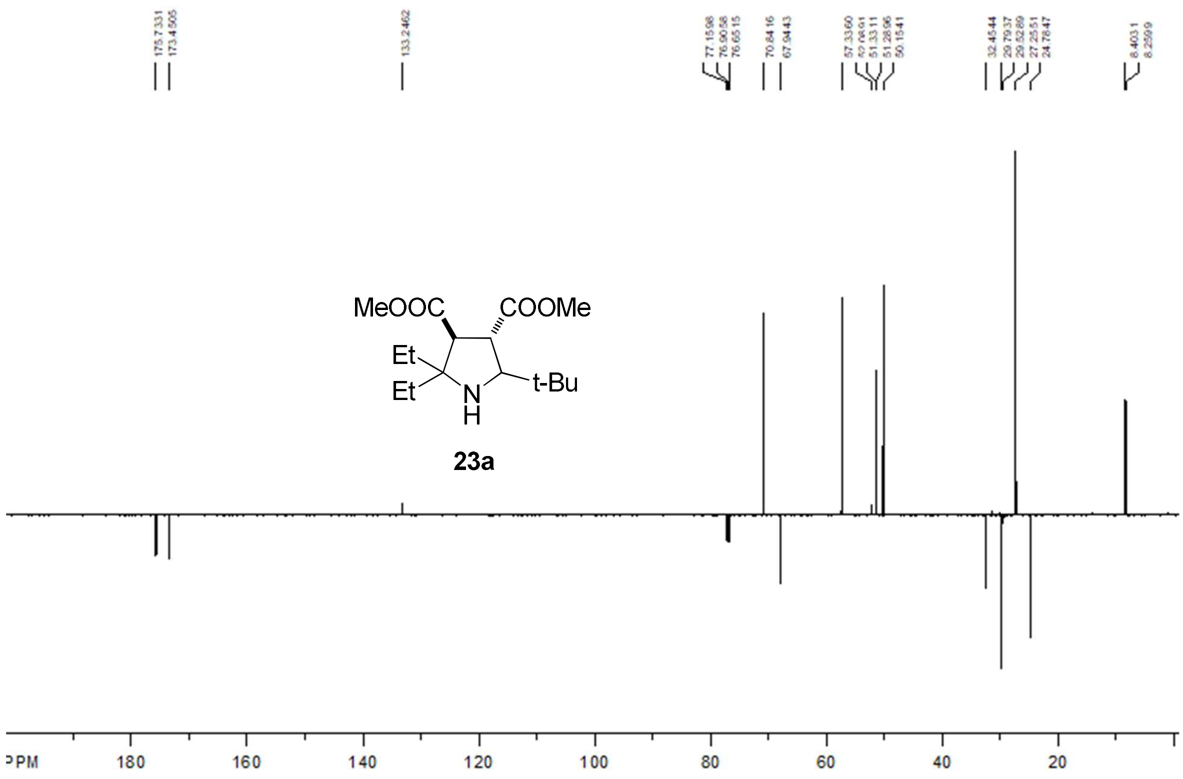

Figure SI53. ^{13}C NMR spectrum of **23a** in $CDCl_3$ at 75 MHz

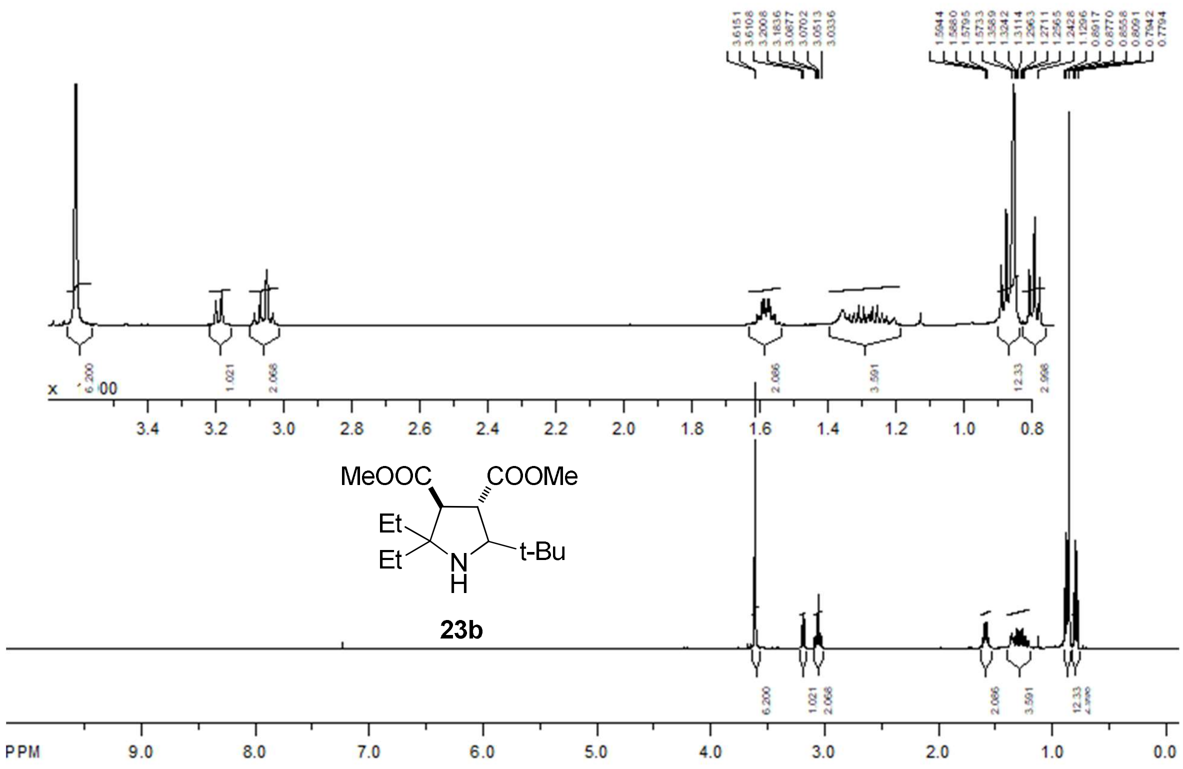

Figure SI54. ^{1}H NMR spectrum of **23b** in $CDCl_3$ at 300 MHz

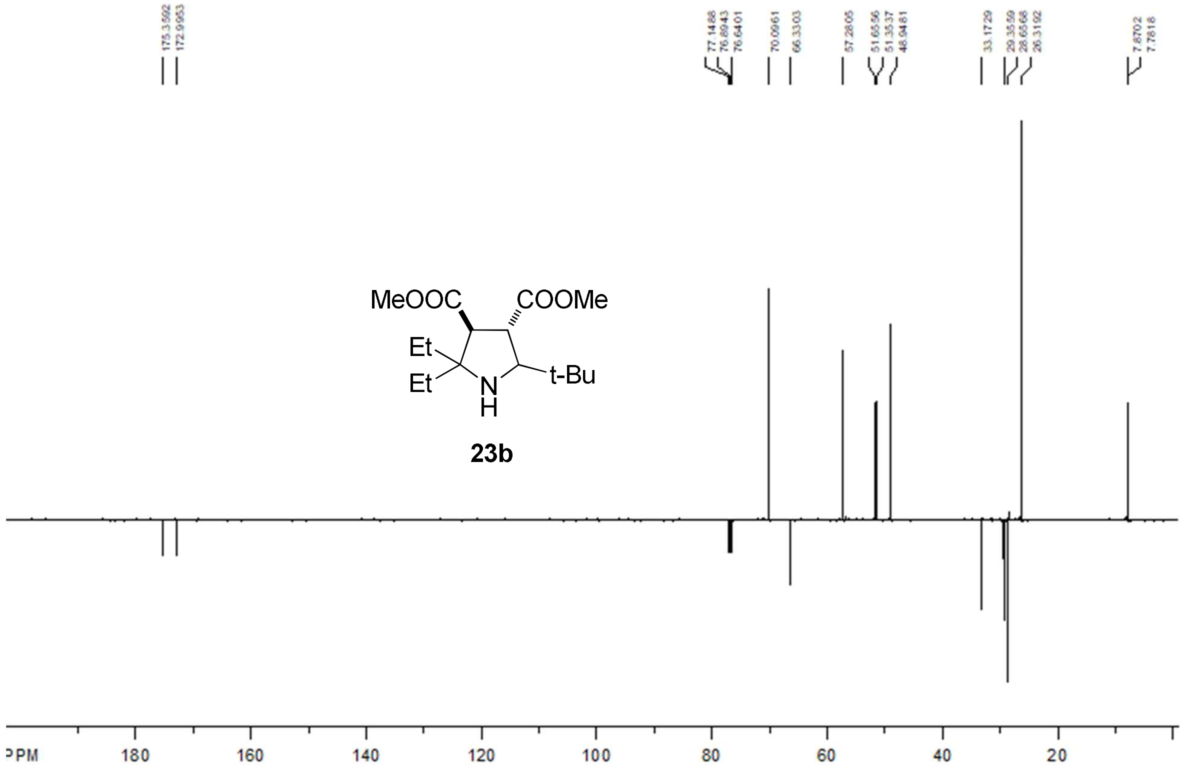

Figure SI55. ^{13}C NMR spectrum of **23b** in $CDCl_3$ at 75 MHz

3.11 5-(*tert*-Butyl)-2,2-diethyl-3,4-bis(hydroxymethyl)-3,4-dihydro-2*H*-pyrrole 1-oxide (**24**)

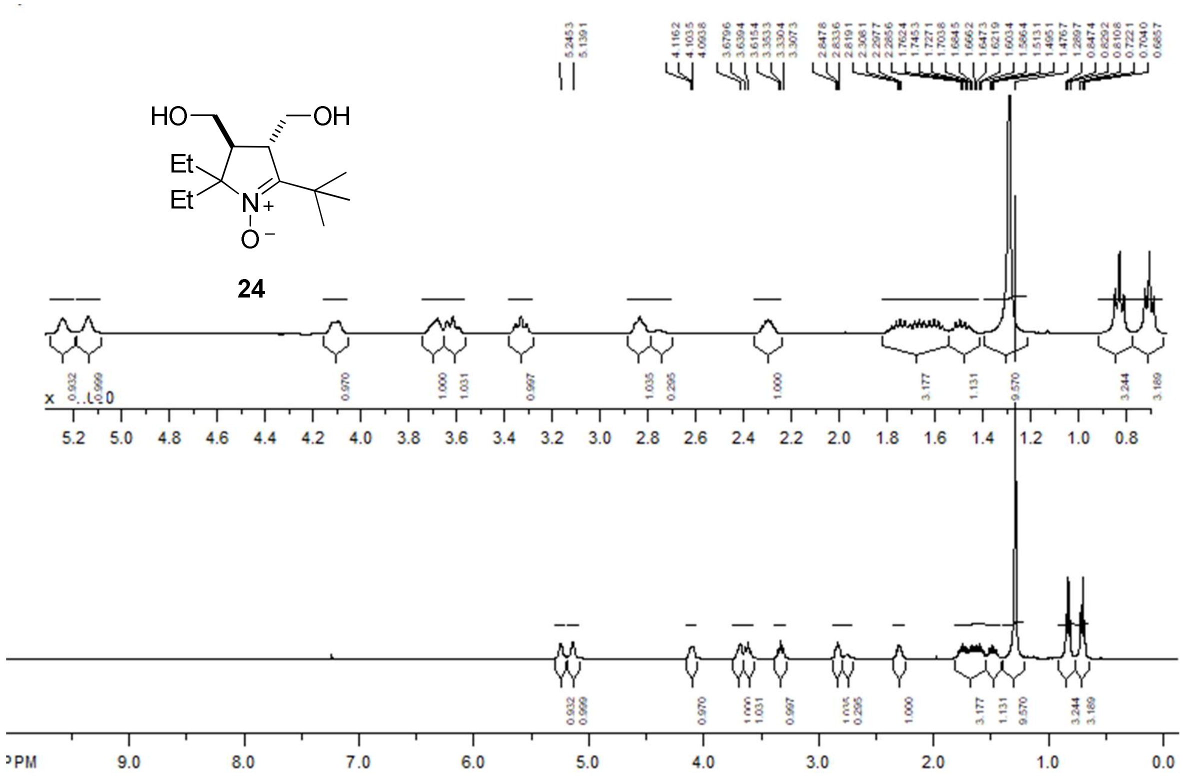

Figure SI56. ^{1}H NMR spectrum of **24** in $CDCl_3$ at 300 MHz

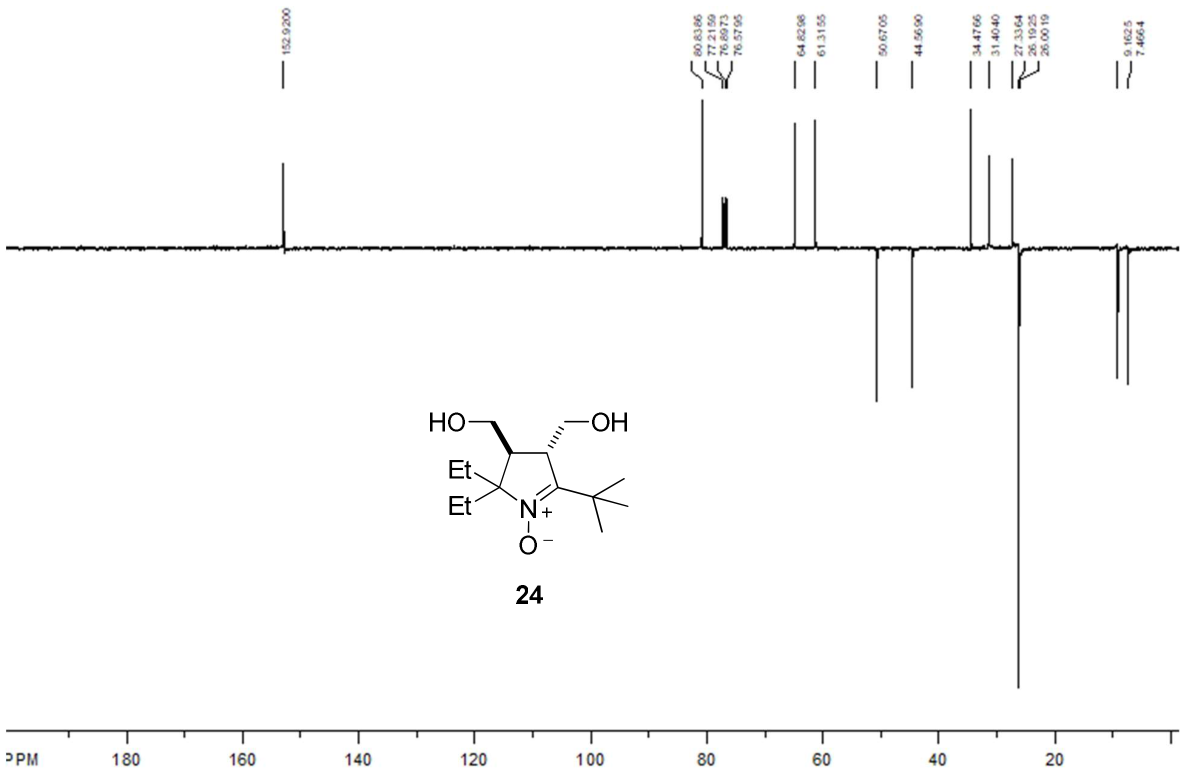

Figure SI57. ^{13}C NMR spectrum of **24** in $CDCl_3$ at 75 MHz

3.12 5-(*tert*-Butyl)-5-butyl-2,2-diethylpyrrolidine-1,3-diol 2,2,2-trifluoroacetate (**4H**)

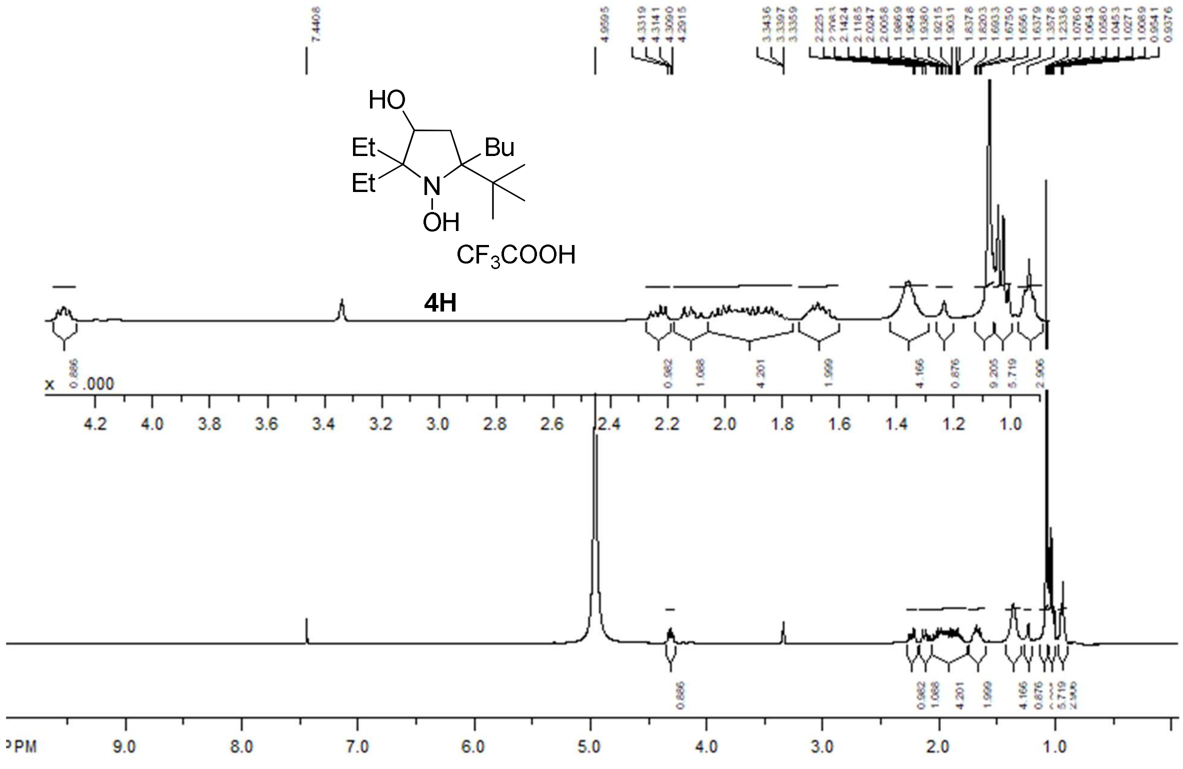

Figure SI58. ^{1}H NMR spectrum of **4H** in $CDCl_3$, CD_3OD, CF_3COOH at 400 MHz

3.13 (2-(*tert*-Butyl)-2-butyl-5,5-diethyl-1-hydroxypyrrolidine-3,4-diyl)dimethanol 2,2,2-trifluoroacetate (**5H**)

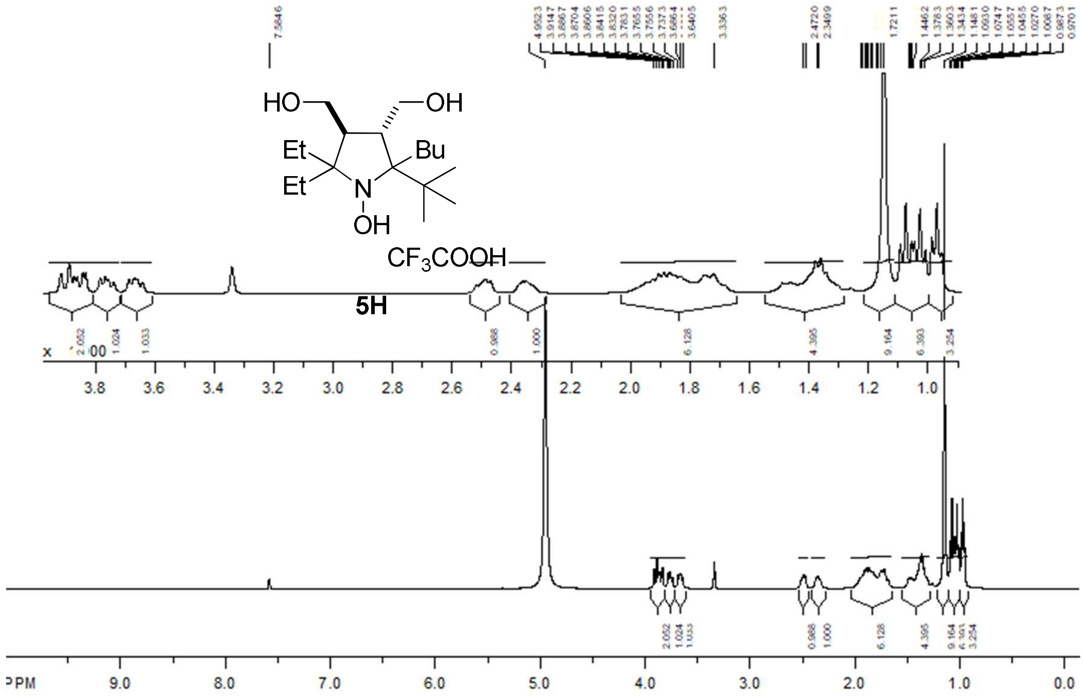

Figure SI59. ^{1}H NMR spectrum of **5H** in $CDCl_3$, CD_3OD, CF_3COOH at 400 MHz

3.14 3-Carboxy-2,2,5,5-tetraethyl-1-hydroxypyrrolidin-1-ium 2,2,2-trifluoroacetate (**1H**)

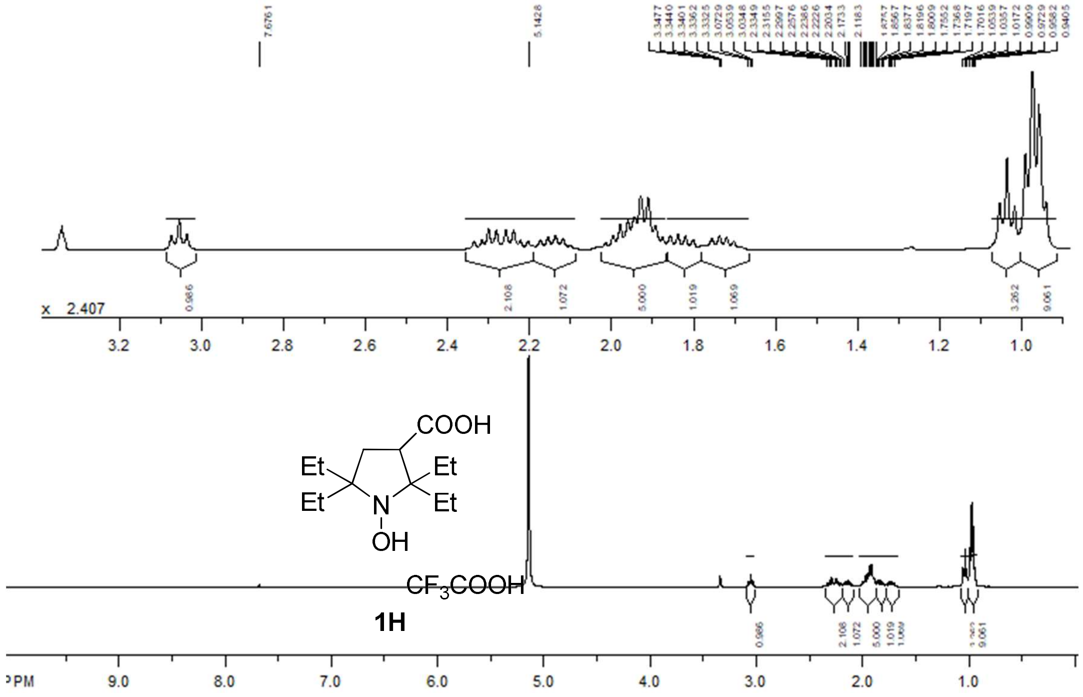

Figure SI60. ^{1}H NMR spectrum of **1H** in $CDCl_3$, CD_3OD, CF_3COOH at 400 MHz

3.15 (3S,4S)-2,2,5,5-Tetraethyl-1-hydroxy-3,4-bis(hydroxymethyl)pyrrolidin-1-ium 2,2,2-trifluoroacetate (**2H**)

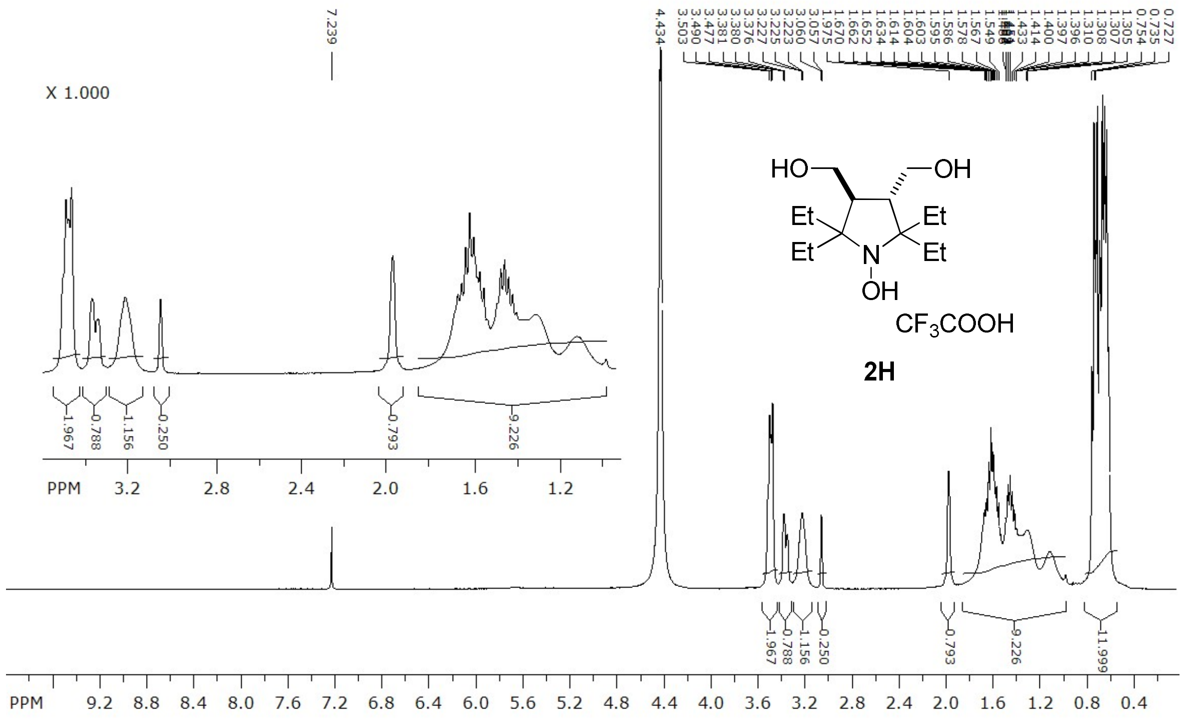

Figure SI61. ^{1}H NMR spectrum of **2H** in $CDCl_3$, CD_3OD, CF_3COOH at 400 MHz

4. 1D and 2D NMR spectra used for structure determination of compound **26** (5-(*tert*-butyl)-2,2-diethyl-3-(hydroxymethyl)-4-pentyl-3,4-dihydro-2*H*-pyrrole)

4.1 ^{1}H NMR spectrum of **26**.

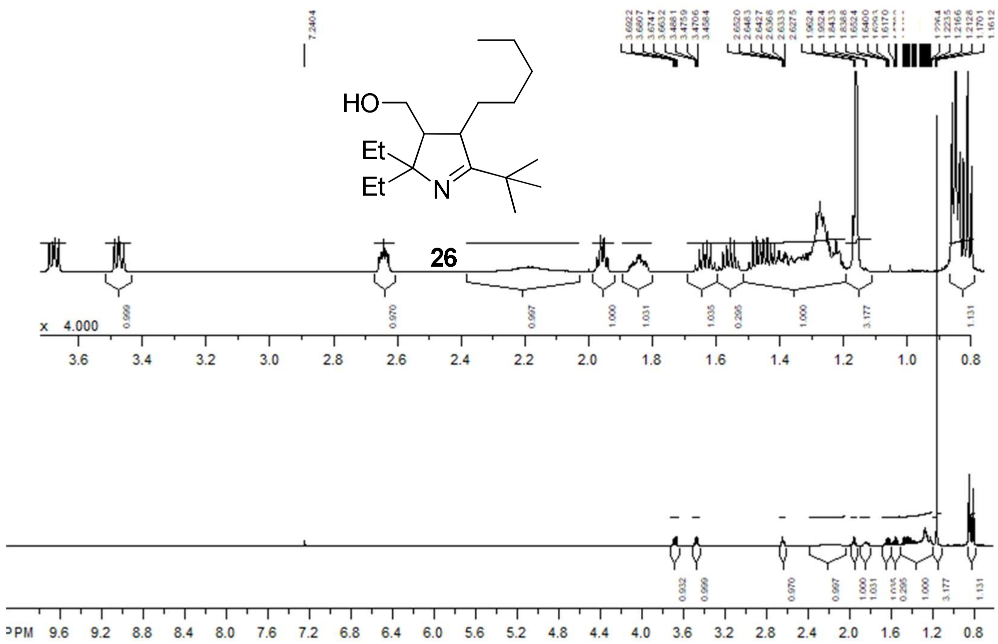

Figure SI62. ^{1}H NMR spectrum of **26** in $CDCl_3$ at 600 MHz

4.2 ^{13}C NMR spectrum of **26**.

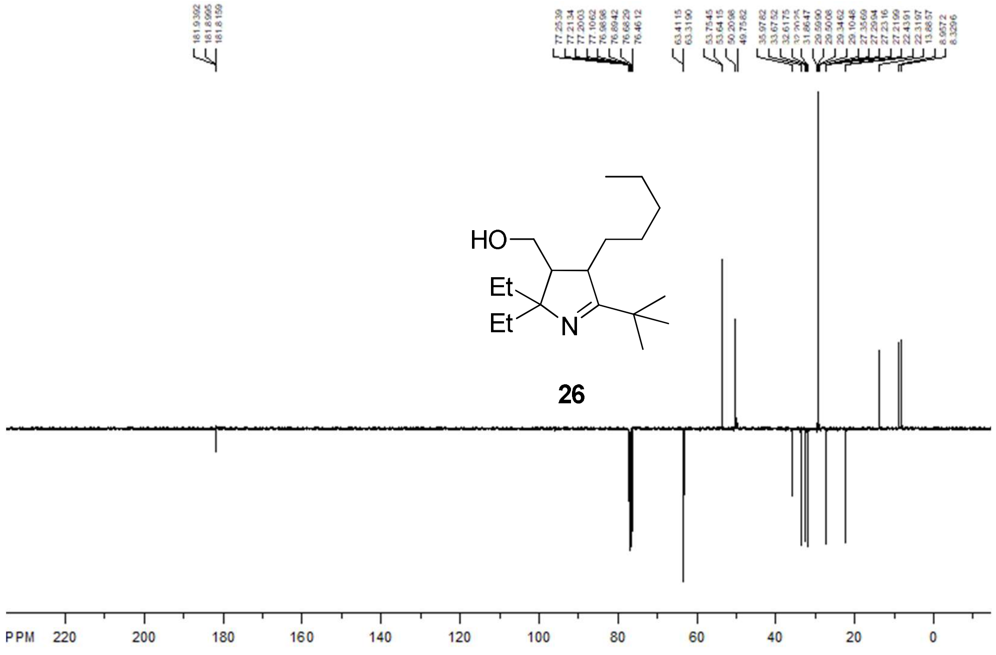

Figure SI63. ^{13}C NMR spectrum of **26** in $CDCl_3$ at 150 MHz

4.3 ^{1}H-^{1}H (COSY) NMR spectrum of **26**.

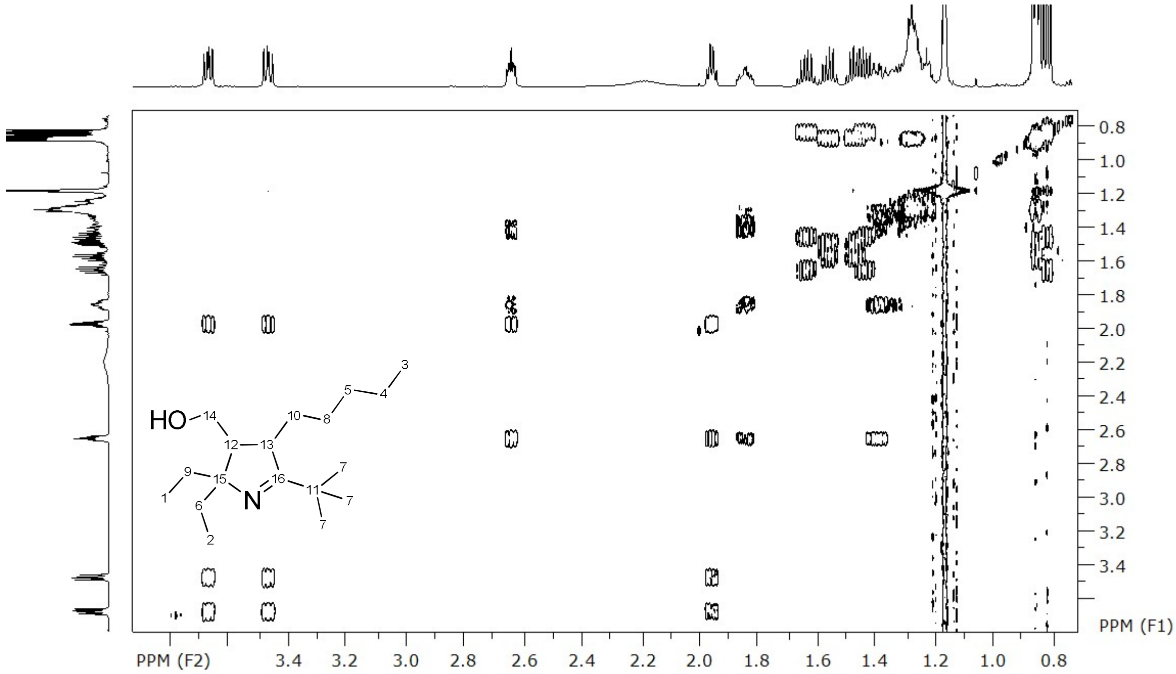

Figure SI64. ^{1}H-^{1}H NMR (COSY) spectrum of **26** in $CDCl_3$ at 600 MHz

4.4 ^{1}H-^{13}C (HSQC) NMR spectrum of **26**.

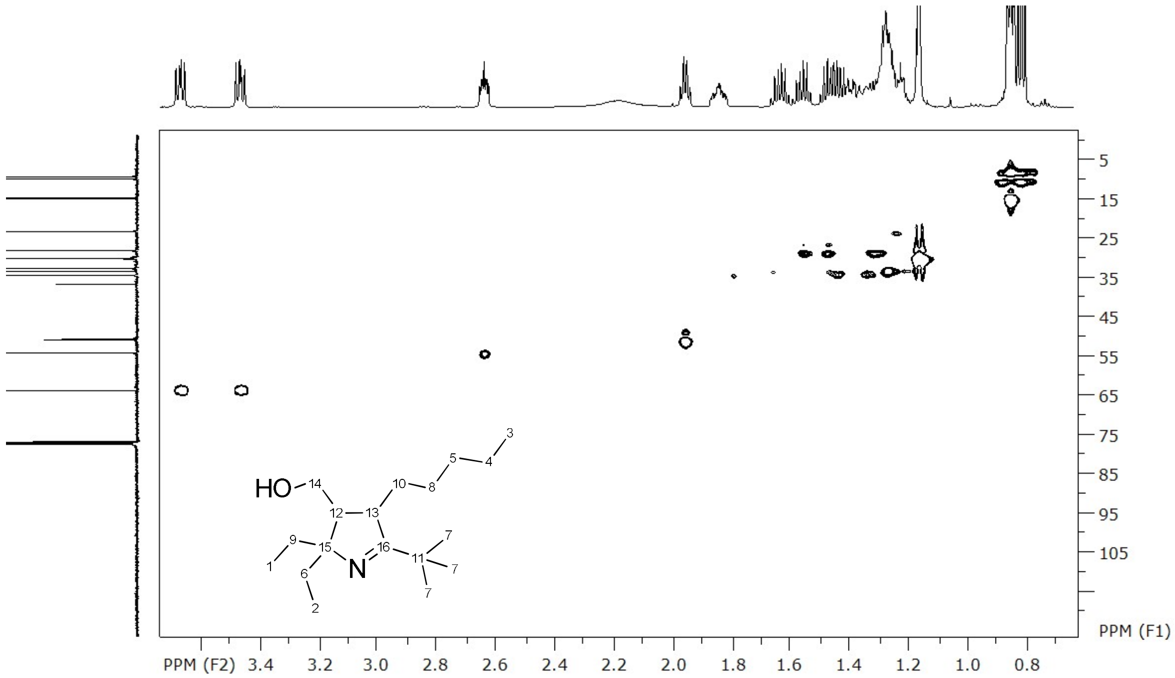

Figure SI65. ^{1}H-^{13}C NMR (HSQC) spectrum of **26** in $CDCl_3$ at 150 MHz

4.5 ^1H-^{13}C (HMBC) NMR spectrum of **26**.

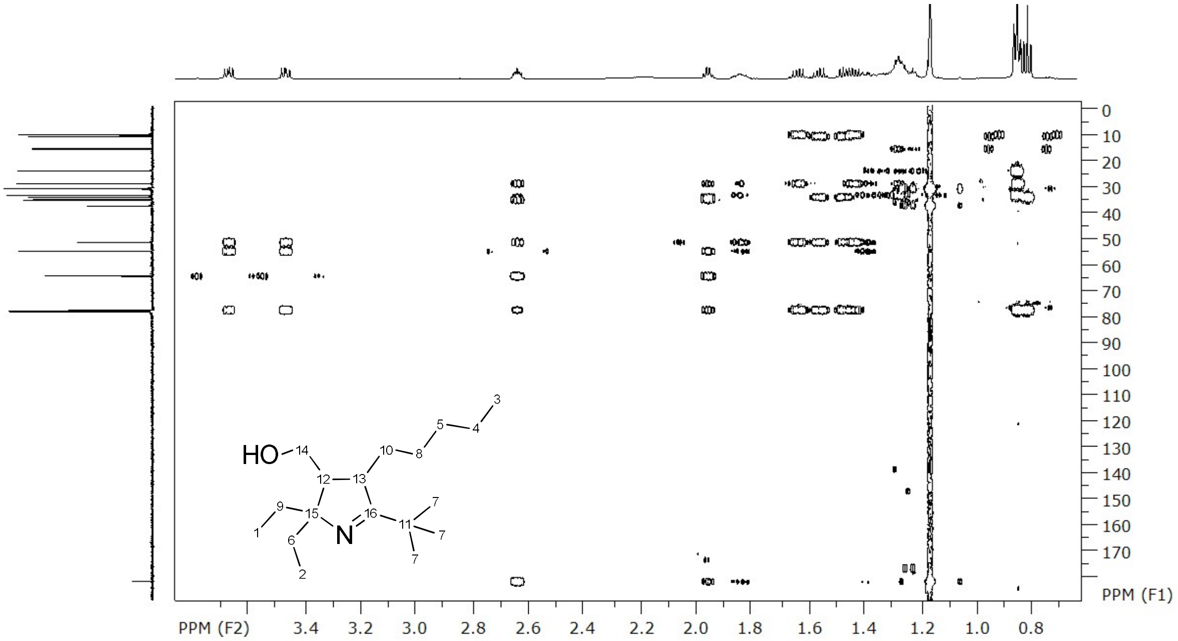

Figure SI66. ^1H-^{13}C NMR (HMBC) spectrum of **26** in $CDCl_3$ at 150 MHz

In ^{13}C NMR spectra of compound **26** recorded in J-modulation mode, 16 signals of carbon atoms are observed (see **Table SI1**): 3 signals for the methyl groups (1, 2 and 3), 2 signals for *tert*-butyl (7,11), 6 - for CH_2 moieties (4,5,6,8,9,10), 2 - for CH fragments (12, 13), signal for CH_2 at heteroatom (14), nodal carbon signal (15) and imine carbon signal (16). One CH_2 signal at the heteroatom disappeared, the signal of the nitrone group is shifted to a weaker field, whereas four additional signals of CH_2 fragments and the signal of the methyl group appeared as compared to the ^{13}C spectra of initial nitrone **24**. Considering the product formation in a reaction with *n*-butyllithium it can be assumed that an addition of *n*-butyl group, as well as deoxigenation of the nitrone and elimination of one of the hydroxy groups occurred.

Analysis of the 2D HSQC spectrum made it possible to unambiguously match the signals of hydrogen atoms in the ^1H spectrum to the signals of carbon atoms in the ^{13}C spectrum for hydroxymethyl group (14), CH fragments (12, 13) at positions 3 and 4 of the heterocycle, as well as for *tert*-butyl group (7).

The following correlations were determined from the 2D COZY spectrum: cross peaks are observed between the signals of hydrogen atoms of hydroxymethyl fragment (14) and CH moiety (12) at 1.92 ppm. CH signal (12), in turn, has a cross peak with a signal of the second CH fragment (13) at 2.61 ppm. The latter also has cross peaks with 1 hydrogen atom signal in the multiplet at 1.35-1.42 ppm and another one at 1.81 ppm (10), which, in turn, has a cross-peak with the signal of the hydrogen atom from the

multiplet region of 1.30-1.35 ppm. Further analysis of this spin system from the COZY spectrum is difficult due to the strong overlap of the signals resulting in a low resolution.

Location of hydroxymethyl group at position 4 of the heterocycle is indicated by cross peaks between the signals of hydroxymethyl hydrogen atoms (14) and the signals of CH carbon atoms (12, 13), as well as the signal of the nodal carbon atom (15) in the 2D HMBC spectrum of **26**. This fact is also confirmed by the presence of cross peaks between the signals of methylene hydrogen atoms of ethyl groups (9) and the signal of CH carbon atom (12). Due to the higher resolution of the HMBC spectrum the signals of CH_2 (5 and 8) were assigned to the *n*-butyl fragment due to the presence of cross peaks between the signals of methyl hydrogen atoms (3) and the signals of CH_2 carbon atoms (4, 5, and 8). In addition, cross peaks between the signal of hydrogen atom at 1.81 ppm. (10) and the signals of imine (16) and CH (13) carbon atoms, as well as the signals of *n*-butyl CH_2 (5 and 8) can be reliably identified. All of that indicates the presence of pentyl substituent at position 3 of the heterocycle.

Table S1. Assignment of the signals of H and C atoms in NMR spectra of compound **26**.

N	H	C	N	H	C
1	0.78 (t, Jt=7.4, 3H)	8.33	9	1.53 (dq, Jd=14.0, Jq=7.4, 1H)<br>1.44 (dq, Jd=14.0, Jq=7.4, 1H)	32.62
2	0.82 (t, Jt=7.2, 3H)	8.96	10	1.81 (dddd, Jd=13.7,Jd=10.4, Jd=5.7, Jd=3.0, 1H)<br>1.19-1.41(m)	33.68
3	0.82 (t, Jt=6.8, 3H)	13.89	11	–	35.98
7	1.13(s, 9H)	29.35	12	1.92 (ddd, Jd=7.4, Jd=6.9, Jd= 5.4, 1H)	50.21
6	1.61 (dt, Jd=14.2,Jt=7.2, 1H)<br>1.40 (dt, Jd=14.2, Jt=7.2, 1H)	27.30<br>27.35	13	2.61 (ddd, Jd=9.2, Jd= 5.4, Jd= 3.2, 1H)	53.64
5	1.19-1.41(m)		14	3.65 (dd, Jd=10.5, Jd=6.9, 1H)<br>3.44 (ddd, Jd=10.5, Jd=7.4, 1H)	63.32
4	1.19-1.41(m)	22.44	15	–	76.46
8		31.86	16	–	181.81

5. HPLC UV chromatograms of 1, 2, 4 and 5.

5.1 5-(*tert*-Butyl)-5-butyl-2,2-diethyl-3-hydroxypyrrolidin-1-oxyl (**4**)

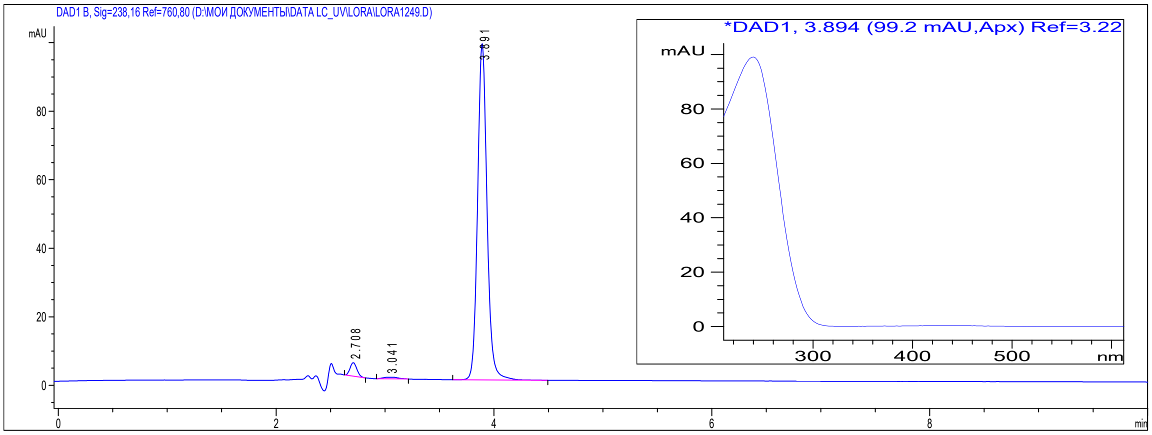

Figure SI67. HPLC chromatogram of **4** and UV spectrum at the main peak (CH_3CN-MeOH-H_2O 20:75:5, 0.5 mg/ml)

Peak	RetTime [min]	Type	Width [min]	Area [mAU*s]	Height [mAU]	Area, %
1	2.708	BB	0.0684	16.99924	3.93708	2.7581
2	3.041	MM	0.1699	5.95170	0.583810	0.9657
3	3.891	BB	0.0923	593.38300	98.43210	96.2762

5.2 2-(*tert*-butyl)-2-butyl-5,5-diethyl-3,4-bis(hydroxymethyl)pyrrolidin-1-oxyl (**5**).

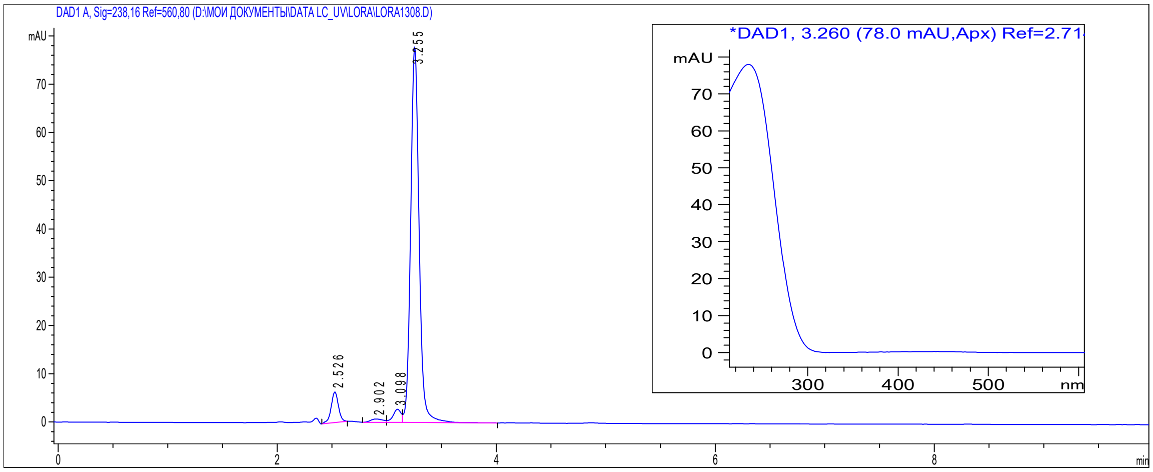

Figure SI68. HPLC chromatogram of **5** before purification and UV spectrum at the main peak (MeOH-H_2O 9:1, 0.6 mg/ml)

Peak	RetTime [min]	Type	Width [min]	Area [mAU*s]	Height [mAU]	Area, %
1	2.526	MM	0.0759	28.69219	6.29792	6.2320
2	3.098	FM	0.0876	14.444408	2.74894	3.1373
3	3.255	VB	0.0825	417.26440	77.92121	90.6307

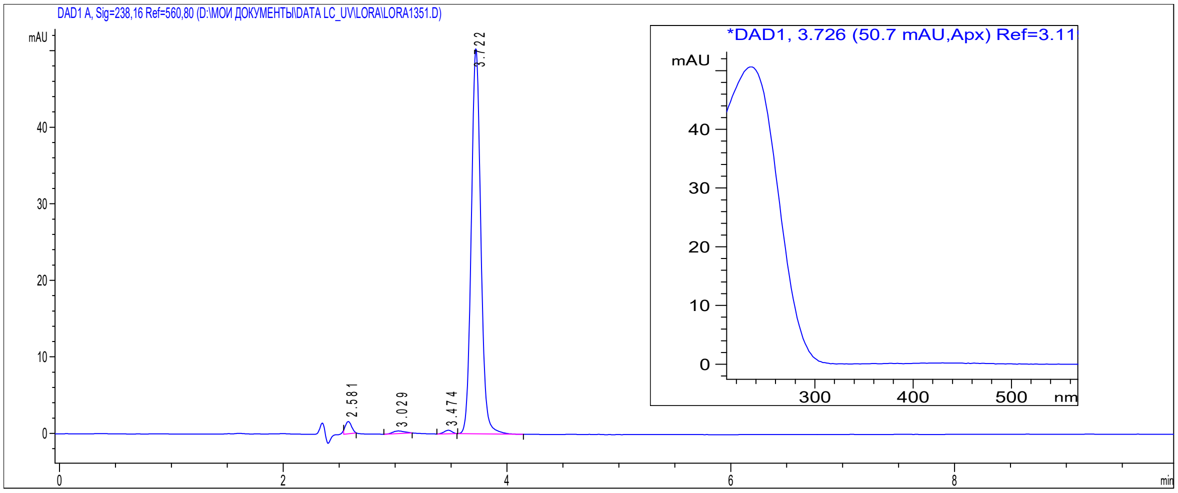

Figure SI69. HPLC chromatogram of **5** after purification and UV spectrum at the main peak (CH_3CN-H_2O 9:1, 0.6 mg/ml)

Peak	RetTime [min]	Type	Width [min]	Area [mAU*s]	Height [mAU]	Area, %
1	2.581	MM	0.0714	7.09532	1.65689	2.3049
2	3.474	BV	0.0814	2.59135	0.492408	0.8418
3	3.722	VB	0.0909	298.14993	50.47675	96.8533

5.3 3-Carboxy-2,2,5,5-tetraethylpyrrolidin-1-oxyl (**1**).

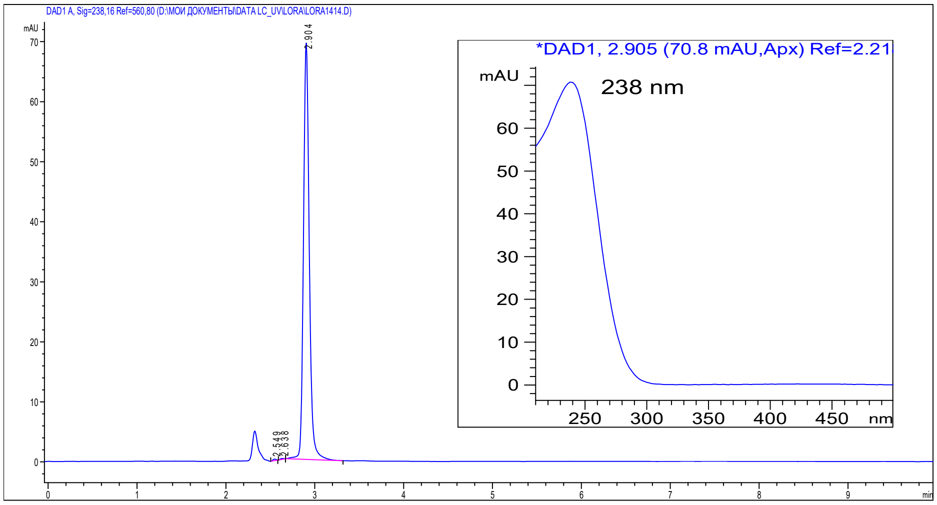

Figure SI70. HPLC chromatogram of **1** and UV spectrum at the main peak (CH_3OH-CH_3CN-H_2O 90:8:2, 0.6 mg/ml); the peak at 2.326 min retention belongs to the solvent (acetonitrile).

Peak	RetTime [min]	Type	Width [min]	Area [mAU*s]	Height [mAU]	Area, %
1	2.549	BB	0.0413	0.653366	0.259771	0.2071
2	2.638	BB	0.0479	0.388598	0.133558	0.1232
3	2.904	BB	0.0709	314.43674	69.36359	99.6697

5.4 2,2,5,5-Tetraethyl-3,4-bis(hydroxymethyl)pyrrolidin-1-oxyl (**2**)

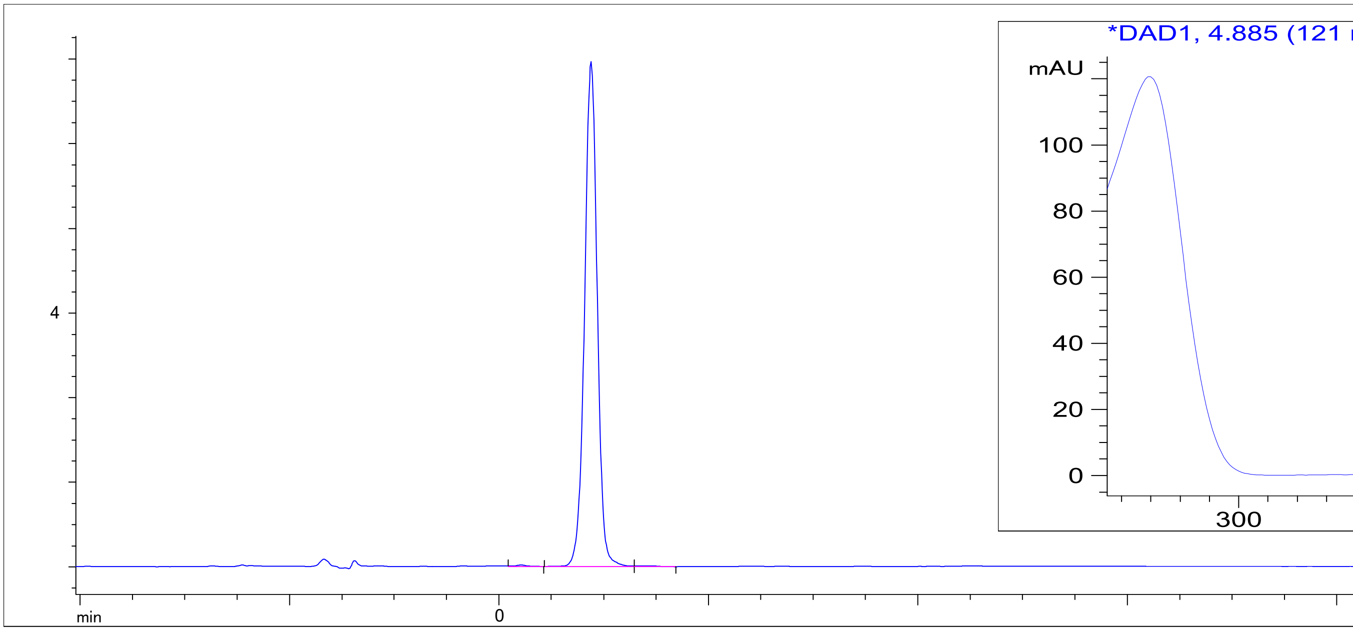

Figure SI71. HPLC chromatogram of **2** and UV spectrum at the main peak (CH_3OH-CH_3CN-H_2O 79:20:1, 0.6 mg/ml)

Peak	RetTime [min]	Type	Width [min]	Area [mAU*s]	Height [mAU]	Area, %
1	4.213	VB	0.1231	3.12452	0.383274	0.3126
2	4.879	BV	0.1271	993.47113	119.35629	99.4009
3	5.420	VB	0.1819	2.86372	0.232107	0.2865

6. X-ray analysis data for compounds 4, 5 and 12.

6.1 Experimental details for compounds **4**, **5**, **12**.

Table S2. Experimental details for compounds **4**, **5**, **12**

	**4**	**5**	**12**
Crystal data			
Chemical formula	$C_{16}H_{32}NO_2$	$C_{18}H_{36}NO_3$	$C_{24}H_{38}N_2O_4$
M_r	270.42	314.48	418.56
Crystal system, space group	Orthorhombic, $Pca2_1$	Monoclinic, $P2_1/c$	Orthorhombic, $P2_12_12_1$
Temperature (K)	200(2)	200(2)	296(2)
a, b, c (Å)	30.038(2), 6.7234(3), 16.3246(9)	9.3844(4), 16.1550(8), 12.8647(6)	8.939(2), 15.522(2), 17.667(3)
α, β, γ (°)	90, 90, 90	90, 107.747(2), 90	90, 90, 90
V (Å^3)	3296.8(3)	1857.5(2)	2451.1(7)
Z	8	4	4
F(000)	1208	700	912
D_x (Mg m^{-3})	1.090	1.124	1.134
μ (mm^{-1})	0.07	0.08	0.08
Crystal size (mm)	0.90 × 0.20 × 0.04	0.75 × 0.50 × 0.10	0.50 × 0.22 × 0.22
Data collection			
T_{min}, T_{max}	0.748, 0.970	0.701, 0.746	0.980, 0.988
No. of measured, independent and observed [$I > 2\sigma(I)$] reflections	29704, 7443, 5303	23153, 4264, 3528	3032, 3032, 2262
R_{int}	0.075	0.024	0.063
θ values (°)	2.5 – 27.5	2.5 – 27.5	2.3 – 28.5

Range of h, k, l	$-39 \leq h \leq 39$, $-8 \leq k \leq 8$, $-20 \leq l \leq 21$	$-12 \leq h \leq 12$, $k = -20 \leq$ ≤ 20, $l = -16 \leq \leq 16$	$-12 \leq h \leq 12$, $0 \leq k \leq 19$, $0 \leq l \leq 22$
Refinement			
$R[F^2 > 2\sigma(F^2)]$, $wR(F^2)$, S	0.051, 0.117, 1.01	0.053, 0.148, 1.02	0.049, 0.136, 1.02
No. of reflections	7443	4264	3032
No. of parameters	355	211	271
No. of restraints	1	0	0
$\Delta\rho_{max}$,$\Delta\rho_{min}$ (e Å^{-3})	0.19, -0.23	0.56, -0.17	0.14, -0.16

6.2 The structure and atom numbering of **4**.

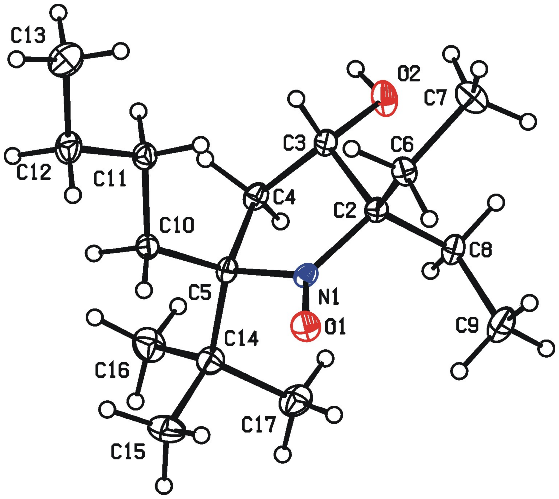

Fig. SI72. The structure and atom numbering of **4** (The thermal ellipsoids are drawn at the 30% probability level).

6.3 The structure and atom numbering of **5**.

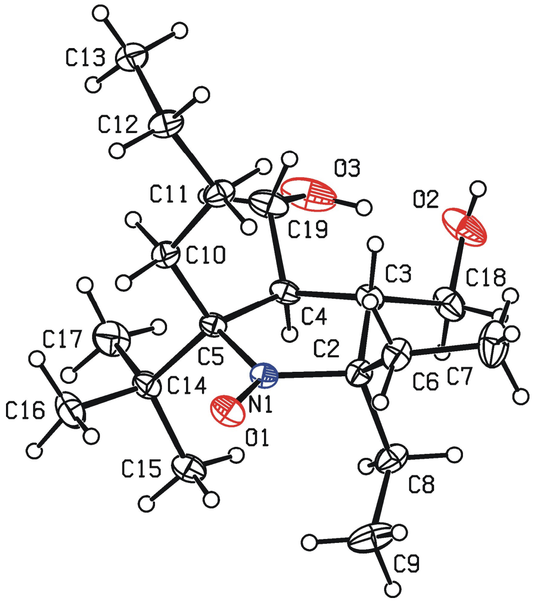

Fig. SI73. The structure and atom numbering of **5** (The thermal ellipsoids are drawn at the 30% probability level).

6.4 The structure and atom numbering of **12**.

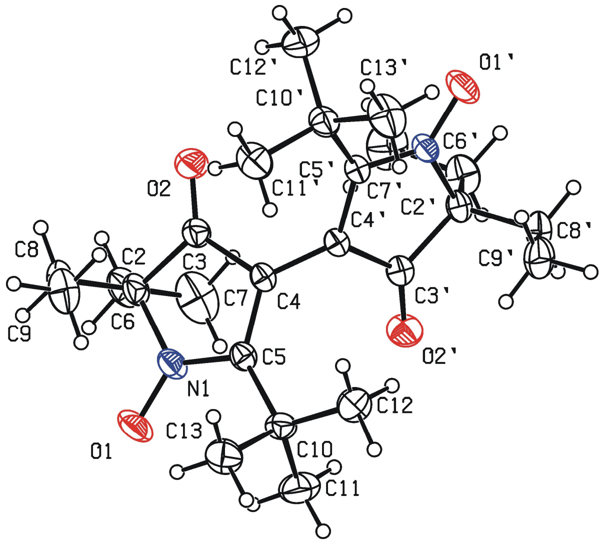

Fig. SI74. The structure and atom numbering of **12** (The thermal ellipsoids are drawn at the 30% probability level).

7. EPR spectral data.

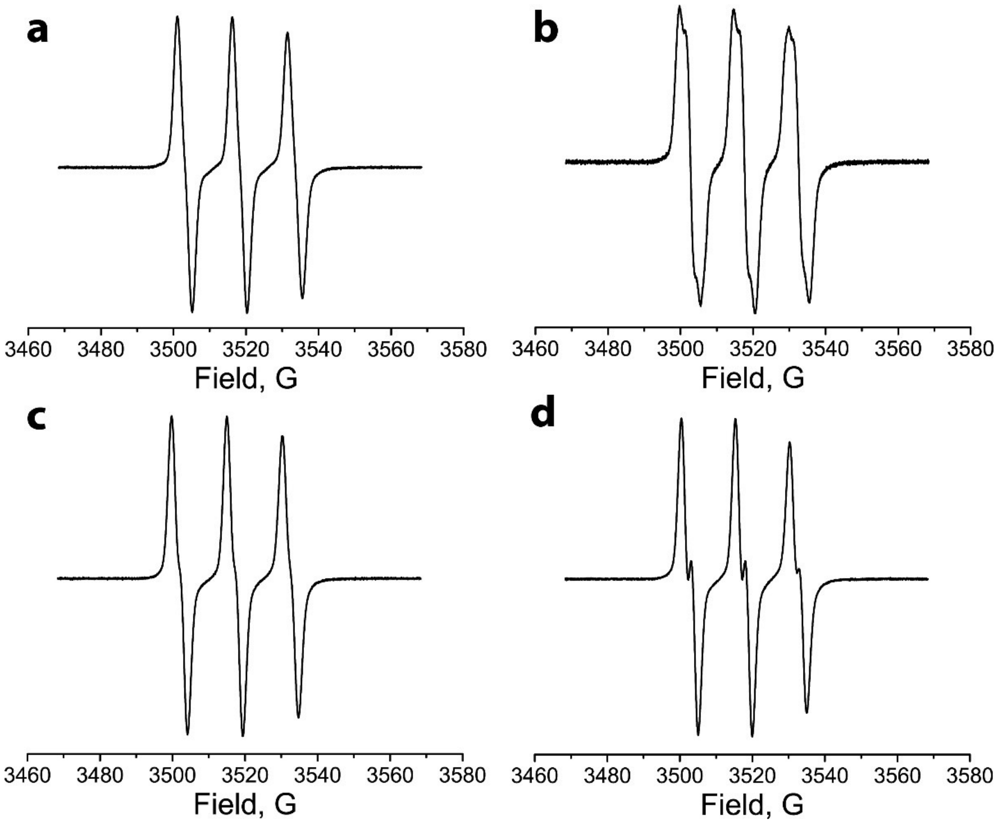

Fig. SI75. EPR spectra of nitroxides **1Na** (a), **2** (b), **4** (c) and **5** (d) in 0.4 mM in H_2O-CH_3OH 1:1 at 293 K, pH 7.4; spectrometer parameters: frequency, 9.87 GHz; microwave power, 2.0 mW; modulation amplitude, 1.5 G; time constant, 10.24 ms; conversion time, 10.24 ms, sweep range 10 mT, scan time 82.52 ms.

8. Reduction kinetics data

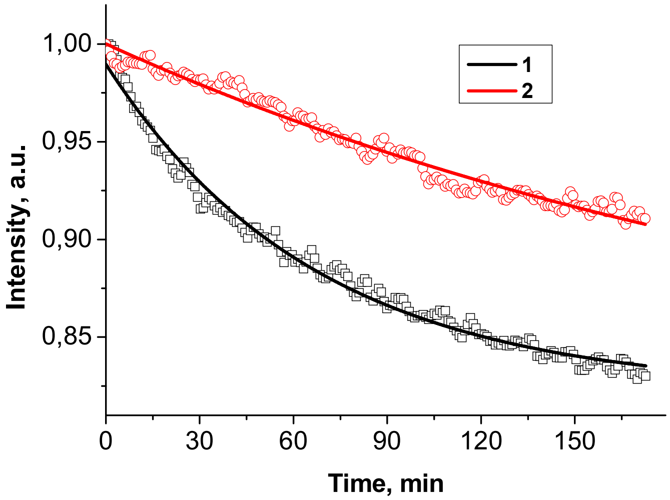

Fig. SI76. Decay of nitroxides **1** and **2** in 50 mM PBS (pH 7.4) at 293 K in sealed capillary; reagents concentrations: nitroxide, 0.3 mM; glutathione, 2 mM; ascorbate, 166.7 mM.

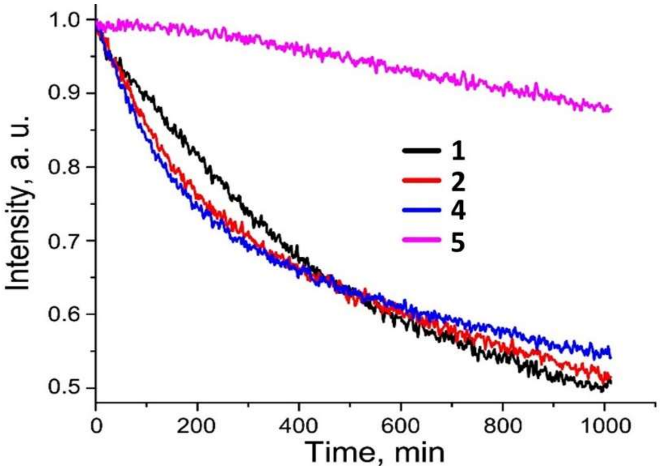

Fig. SI77. Kinetics of **1**, **2**, **4** and **5** decay in the presence of 0.5 M ascorbate in 50% methanol at pH 7.4.

9. Thermal stability of nitroxides 4 and 5.

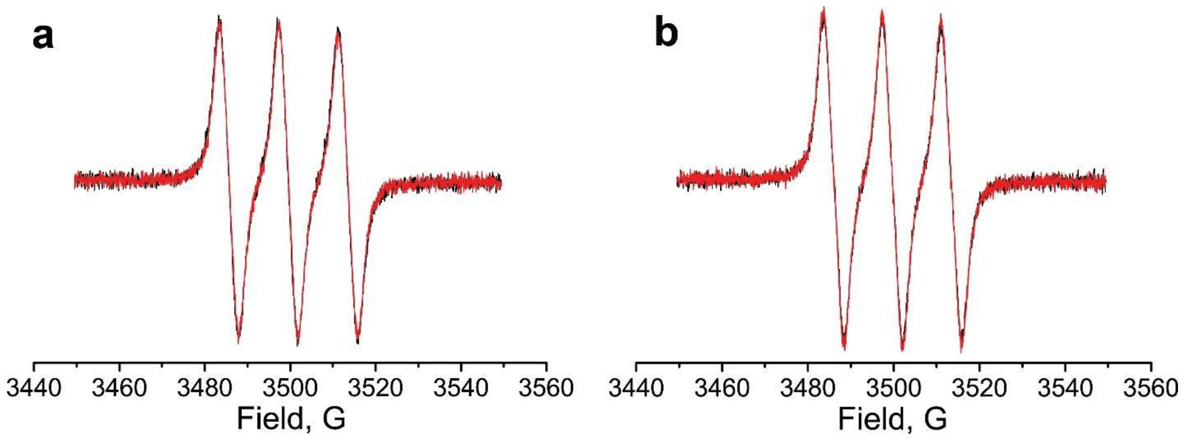

Fig. SI78. The EPR spectra of **4** (**a**) and **5** (**b**) in toluene before (**black**) and after (**red**) heating to 95-100 °C for 80 min.
